# Molecular subtyping and the construction of a predictive model of colorectal cancer based on ion channel genes

**DOI:** 10.1186/s40001-024-01819-2

**Published:** 2024-04-04

**Authors:** Lian-jie Ai, Guo-dong Li, Gang Chen, Zi-quan Sun, Jin-ning Zhang, Ming Liu

**Affiliations:** 1 Colorectal Tumor Surgery, The 2nd Affiliated Hospital of Harbin Medical University, Harbin, 150001 Heilongjiang China; 2 General Surgery, The 4th Affiliated Hospital of Harbin Medical University, Harbin, 150001 Heilongjiang China

**Keywords:** Ion channel, Colorectal cancer, Prognosis, Immune infiltration, Immunotherapy, Drug sensitivity

## Abstract

**Purpose:**

Colorectal cancer (CRC) is a highly heterogeneous malignancy with an unfavorable prognosis. The purpose of this study was to address the heterogeneity of CRC by categorizing it into ion channel subtypes, and to develop a predictive modeling based on ion channel genes to predict the survival and immunological states of patients with CRC. The model will provide guidance for personalized immunotherapy and drug treatment.

**Methods:**

A consistent clustering method was used to classify 619 CRC samples based on the expression of 279 ion channel genes. Such a method was allowed to investigate the relationship between molecular subtypes, prognosis, and immune infiltration. Furthermore, a predictive modeling was constructed for ion channels to evaluate the ion channel properties of individual tumors using the least absolute shrinkage and selection operator. The expression patterns of the characteristic genes were validated through molecular biology experiments. The effect of potassium channel tetramerization domain containing 9 (KCTD9) on CRC was verified by cellular functional experiments.

**Results:**

Four distinct ion channel subtypes were identified in CRC, each characterized by unique prognosis and immune infiltration patterns. Notably, Ion Cluster3 exhibited high levels of immune infiltration and a favorable prognosis, while Ion Cluster4 showed relatively lower levels of immune infiltration and a poorer prognosis. The ion channel score could predict overall survival, with lower scores correlated with longer survival. This score served as an independent prognostic factor and presented an excellent predictive efficacy in the nomogram. In addition, the score was closely related to immune infiltration, immunotherapy response, and chemotherapy sensitivity. Experimental evidence further confirmed that low expression of KCTD9 in tumor tissues was associated with an unfavorable prognosis in patients with CRC. The cellular functional experiments demonstrated that KCTD9 inhibited the proliferation, migration and invasion capabilities of LOVO cells.

**Conclusions:**

Ion channel subtyping and scoring can effectively predict the prognosis and evaluate the immune microenvironment, immunotherapy response, and drug sensitivity in patients with CRC.

**Supplementary Information:**

The online version contains supplementary material available at 10.1186/s40001-024-01819-2.

## Introduction

Colorectal cancer (CRC) is the second deadliest and third most prevalent malignant tumor worldwide [1]. It arises from the epithelial cells of the colon or rectal mucosa and is a multifactorial disease involving multiple genes and stages [1]. The incidence and mortality rates of CRC are continuously increasing globally. CRC is often detected in the intermediate to advanced stages due to its elusive onset and lack of typical early symptoms [2]. Despite recent advancements in prognostic models [3, 4] and treatment options, the patient prognosis remains unsatisfactory. The biology of CRC and its sensitivity to treatment vary among patients due to its high heterogeneity and complexity [5]. Therefore, accurate patient stratification is crucial to accurately predict prognosis and develop targeted treatment strategies [6].

Ion channels are a class of proteins involved in the regulation of ion flow across cell membranes. They not only play a role in various physiological processes, but also are key regulators in the pathophysiology of cancer cells [7], including carcinogenesis [8], proliferation [9], migration [10], and drug resistance [11]. As shown in a recent study, the abnormal expression of ion channels is closely associated with the occurrence and development of CRC. For instance, the voltage-gated potassium channel Kv1.3 is abnormally expressed in CRC [12]; the sodium channel epithelial 1 subunit beta inhibits the occurrence of CRC by suppressing the active c-Raf and mitogen-activated protein kinase signals [13]; the voltage-gated sodium channel Nav1.5 is a key regulator of the gene transcription network controlling colon cancer invasion [14]; low expression of chloride channel accessory 1 indicates a poor prognosis in CRC [15]. Furthermore, drugs targeting ion channels have been found to reverse the drug-resistant properties of CRC. For example, riluzole, an ion channel modulator, can reduce the resistance of CRC cells to cisplatin [16]; the sodium channel Nav1.5 encoded by sodium voltage-gated channel alpha subunit 5 enhances the chemical sensitivity of CRC cells to 5-fluorouracil [17]. In addition, ion channels are abundantly expressed in immune cells and play an important role in maintaining immune cell activity, regulating immune responses, and modulating communication between tumors and immune cells [18, 19]. For instance, the ion channel of the transient receptor potential cation channel subfamily V member 1 gain-of-function reprograms the immune microenvironment to promote colorectal tumorigenesis [20]; inactivation of calcium ion channels that recognize tumor antigens can affect the anti-cancer immunity of tumor cells, and the hyperpolarization of tumor cell can increase the likelihood of immune evasion and promote tumor progression [21]. These findings suggested ion channels have great potential as diagnostic markers, prognostic indicators, and therapeutic targets in tumors. However, further studies are needed to fully understand the molecular subtypes and develop prognostic models based on ion channel genes in CRC.

In this study, CRC samples from The Cancer Genome Atlas (TCGA) were classified based on ion channel genes, revealing significant differences in survival, immune infiltration, and biological functions among the various subtypes. Subsequently, a predictive modeling was constructed based on these ion channel subtypes. The results of this study highlight the potential of the ion channel scoring system as a robust prognostic indicator for CRC. In addition, it can be employed to predict the response to immunotherapy and drug sensitivity, thereby contributing to the selection and development of optimal treatment strategies.

## Materials and methods

### Data set acquisition

The Genome Data Commons was used to download the TCGA CRC gene expression, clinical phenotype, and survival data. The information about TCGA CRC mutations was provided by the Genome Data Commons. Imvigor210 served as an immunotherapy dataset derived from the study of Mariathasan et al. [22]. The CRC prognostic model utilized for comparison was generated from the published studies [23–29]. The tumor immune dysfunction and exclusion (TIDE) score was collected from the official website [30]. A total of 279 ion channel genes were offered by the HUGO Gene Nomenclature Committee and published literature [31, 32].

### Consensus clustering of ion channel genes

The ConsensusClusterPlus package was used to classify 619 TCGA CRC samples based on 279 ion channel genes.

### Functional and pathway enrichment analysis

The related Kyoto Encyclopedia of Genes and Genomes C2 gene set was obtained from MSigDB and the gene set variation analysis package was utilized for pathway analysis [33]. Gene Ontology enrichment analysis was carried out using the clusterProfiler package.

### Immune cell infiltration analysis

The single-sample gene set enrichment analysis (ssGSEA) was used to calculate an enrichment score for each sample to reflect the degree of immune cell infiltration in the sample [34]. The TIMER official website was performed to retrieve the proportions of 22 immune cell types projected by the cell-type identification using estimating relative subsets of RNA transcripts algorithm to be penetrated in the TCGA samples. Such proportions were also used for downstream immune infiltration comparisons. The immune score and ESTIMATE score were calculated for each sample using the hacksig package and further comparison studies were carried out.

### Differentially expressed genes among ion channel subtypes and consistent clustering analysis

Limma was applied to detect differentially expressed genes (DEGs) between internal and external samples for each ion channel subtype [35]. To perform functional enrichment analysis, the identified DEGs were combined for each ion channel subtype. Three gene subtypes were discovered in the combined DEGs.

### Ion channel prognostic model acquisition and assessment

The initial set was restricted to 19 DEGs, which was significant in univariate Cox regression (*p* < 0.01). The model was constructed using the least absolute shrinkage and selection operator regression (LASSO) [36] and the cross-validated lambda.min corresponding model was used as the final ion channel prognostic model. The ion channel score was calculated as the sum of the product of the model characteristic gene expression and the corresponding model coefficient. Based on the ion channel score, the test set (TCGA CRC) and validation set (GSE38832) cohorts were divided into low- and high-score groups. The survival and survminer packages were used for log-rank testing and survival curve plotting. The survivalROC package was used to generate receiver operating characteristic curves and the area under the curve values for ion channel models predicting patient survival. The rms package was adopted to analyze and plot nomograms as well as calibration curves. Decision curve analysis was analyzed and visualized using the stdca function. The maftools package was used for mutation landscape plotting.

### Single-cell transcriptome analysis

On the Tisch official website, the cellular taxa was visualized and the expression of genes associated with the ion channel model in each cell population was examined.

### Assessment of drug sensitivity

The pRRophetic package was used to predict the half-maximal inhibitory concentration (IC50) of chemotherapy drugs in each sample [37].

### Tissue specimens

The study was approved by the ethics committee of the Fourth Affiliated Hospital of Harbin Medical University, and the requirement for written informed consent (Approval No. 2022-SCILLSC-37) was waived. The CRC tissue and paracancerous tissue samples were obtained from this committee.

### Real-time quantitative polymerase chain reaction

The levels of mRNA were determined using real-time quantitative polymerase chain reaction (RT-qPCR). In brief, Trizol was used to extract mRNA, the PrimeScriptTM RT Master Mix was utilized for reverse-transcription, and the ChamQ Universal SYBR qPCR Master Mix reagent kit was applied to perform PCR on a LightCycler® 480II device. The relative expressions of the genes were calculated using the equation 2^−ΔΔCt^. Glyceraldehyde-3-phosphate dehydrogenase (GAPDH) served as the internal reference. The primer sequences were as follows: GAPDH (191bp): (forward) 5′-GAAGAGCTACGAGCTGCCTGA-3′ and (reverse) 5′-CAGACAGCACTGTGTTGGCG-3′; potassium channel tetramerization domain containing 9 (KCTD9) (242bp): (forward) 5′-ACCTCCCTACCAATGACT-3′ and (reverse) 5′-ATCTCCTCCCACTATGC-3′ (Additional file 1: Table S1).

### Western blot

Radioimmunoprecipitation assay lysis buffer was used to extract complete proteins from tissues. After loading the same quality of protein, polyacrylamide gel electrophoresis was performed. Then, the proteins were transferred to the polyvinylidine fluoride membrane. Subsequently, the membranes were sealed in 5% blocking solution for 2 h. After that, the membranes were incubated with specific primary antibodies overnight at 4 °C, followed by 1 h of incubation with secondary antibodies at room temperature the next day. The membranes were washed with Tris buffered saline with Tween, and the enhanced chemiluminescence kit was conducted for examination. The obtained protein bands were quantified with densitometry using ImageJ software. β-Actin served as a loading control.

### Immunohistochemistry

Tissue microarrays were prepared from paraffin-embedded CRC and paracancerous tissues [38]. The tissue microarrays were deparaffinized with xylene, the graded ethanol was performed to hydrate the sections, and the thermal repair was applied to carry out antigen repair. Then, the specified primary antibody was added at 4° overnight after blocking with 1% bovine serum albumin solution. The next day, the secondary antibodies were added for 1 h of incubation at 37°. A 3,3′-diaminobenzidine kit was conducted for detection. The immunohistochemistry imaging was performed using a new Olympus SLIDEVIEW VS200 slide scanner and Image J software was used for analysis.

### Cell culture and transfection

The LOVO cells were cultured in Kaighn’s Modification of Ham’s F-12 Medium at 37 °C with 5% CO_2_ humidity, supplemented with 10% fetal bovine serum and 1% penicillin–streptomycin. Next, a comprehensive lentiviral gene transfer method was employed to modify the expression of the KCTD9 gene in the LOVO cell line on a three-plasmid packaging system. The pLVX-CMV-hKCTD9-Puro plasmid was introduced for overexpression, while gene knockdown was achieved through the pLKO-U6-shKCTD9-EGFP-Puro plasmid. The pLKO-U6-shKCTD9-EGFP-Puro plasmid contained specific shRNA sequences targeting KCTD9 (sh-KCTD9-1: GCCAATTTAAGCCGCTGTAAT; sh-KCTD9-2: GCCAATTTAGAAGGTGCTAAT). Both strategies utilized the packaging plasmids psPAX2 and pMD2.G, with 293T cells serving as the lentivirus production hosts. Upon lentivirus generation, the LOVO cells were infected, and the stable integration of the desired genetic modifications was achieved by selecting with 5 μg/ml puromycin.

### Cell counting Kit-8 assay

The Cell Counting Kit-8 (CCK-8) assay was employed to evaluate the viability of the LOVO cells. Initially, cells were seeded in 96-well plates and incubated overnight to ensure adhesion and optimal proliferation. After specific treatments, CCK-8 reagent was added to each well, leveraging its ability to produce a quantifiable colorimetric change via dehydrogenase activity in viable cells. This reaction converts 2-(2-methoxy-4-nitrophenyl)-3-(4-nitrophenyl)-5-(2,4-disulfophenyl)-2*H*-tetrazolium to a soluble formazan dye, and the optimal incubation time was generally 1–4 h to maximize color development. The absorbance at 450 nm was measured, directly correlating with cell viability.

### Colony formation assay

A colony formation assay was conducted to assess the proliferation capacity of LOVO cells. Cells were cultured at low density in 6-well plates and maintained in optimal growth conditions to form colonies within 2 weeks. Upon incubation, the colonies were fixed with methanol and stained with 0.5% crystal violet for visualization and counting.

### Wound healing assay

A wound healing assay was performed to investigate the migratory capabilities of LOVO cells. After the cells reached confluence in 6-well plates, a sterile pipette tip was used to make a uniform scratch on the cell monolayer. Subsequently, cell migration to the wound area was monitored and photographed using an inverted microscope at 0, 24, and 48 h post-scratch. The quantitative analysis of the extent of wound closure indicated cell migration rate.

### Transwell invasion assay

The invasive properties of LOVO cells were evaluated using a Transwell invasion assay. Cells were seeded into the upper chamber of Transwell inserts pre-coated with Matrigel to simulate the extracellular matrix barrier. The lower chamber contained a chemoattractant medium to facilitate cell invasion through the Matrigel. After 24 h of incubation, non-invading cells were gently removed from the upper surface, while cells that migrated to the lower surface of the membrane were fixed, stained with crystal violet, and counted using a microscope.

### Statistical analysis

The Wilcoxon Rank Sum test or *t* test was employed for the comparisons between two groups of successive variables. The Kruskal–Wallis test or analysis of variance was used to compare the successive variables among three or more groups. Comparisons between subtypes were assessed using the Chi-square test or Fisher’s exact test. The statistical analysis of bioinformatics was carried out in the R environment (version 4.1.3).

## Results

### Identification of ion channel subtypes in colorectal cancer

**Fig. 1:**
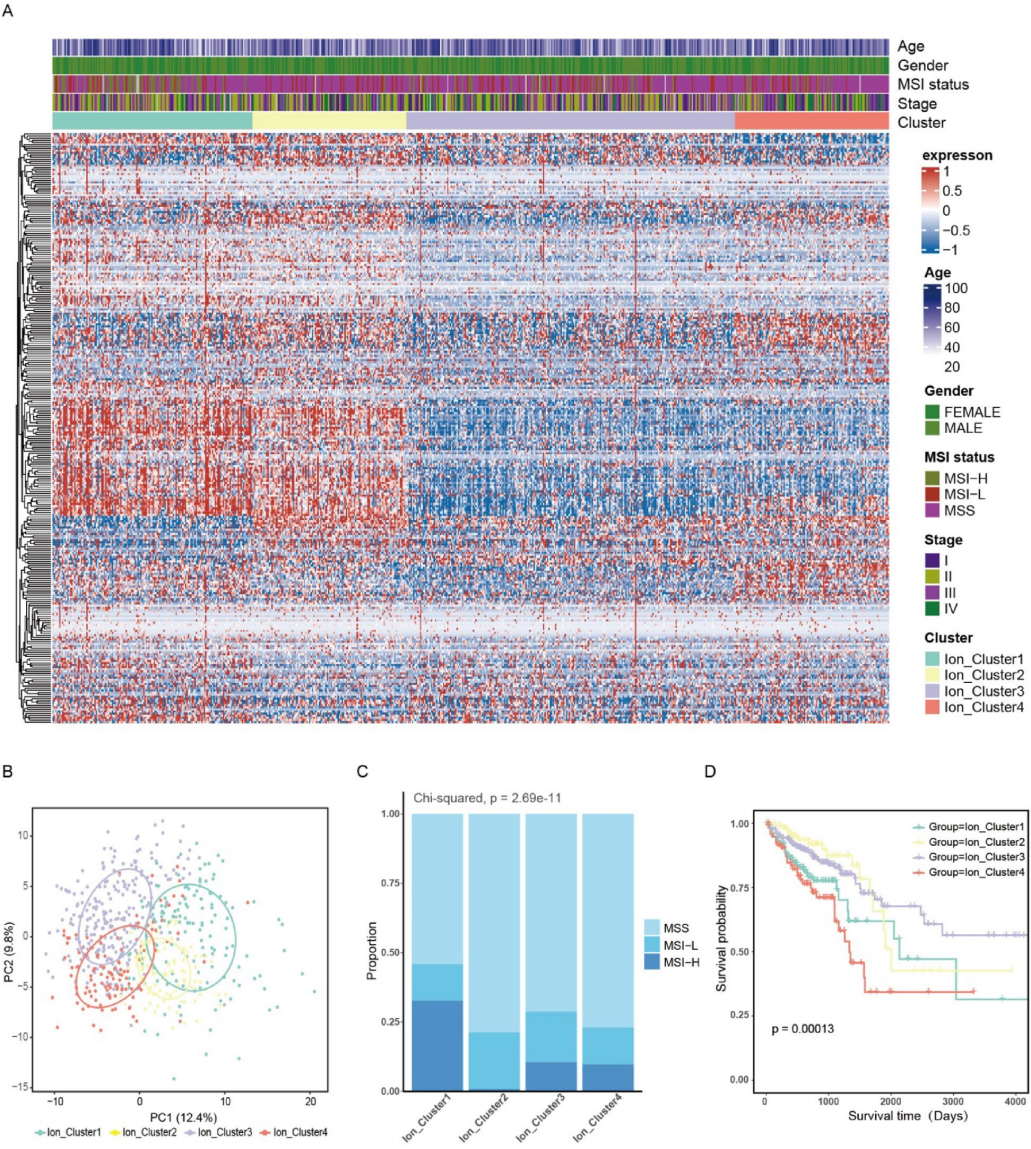
Clinical characteristics of ion channel subtypes in colorectal cancer. **A** The expression of 279 ion channel genes depicted in the TCGA CRC cohorts. The cluster and common clinical variables were used as patient annotations. **B** Four distinct subtypes were identified by principal component analysis of ion channel genes in the TCGA CRC dataset. **C** The proportion of samples (MSS, MSI-L, MSI-H) with microsatellite instability was shown in each subtype. **D** Kaplan–Meier curve among the four subtypes showed significant differences (log-rank test, *p* = 0.00013). *TCGA* The Cancer Genome Atlas, *CRC* colorectal cancer, *MSS* microsatellite stable, *MSI-L* microsatellite instability of lower degree, *MSI-H* microsatellite instability of higher degree

The overall flow of the study was displayed in Additional file 2: Fig. S1. A total of 279 ion channel genes were collected and analyzed by univariate Cox regression, revealing that 36 genes were closely associated with survival (Additional file 2: Fig. S2A). This result highlighted the importance of ion channel genes and their potential for model development. Based on these 279 genes, a consistent clustering method was used to divide 619 CRC samples into four subtypes (Additional file 2: Fig. S2B–D). Each subtype displayed distinct patterns of ion channel expression (Fig. 1A), which were further supported by principal component analysis (Fig. 1B). According to the common clinical variables, we discovered that Ion Cluster1 exhibited the highest proportion of microsatellite instability, particularly high-grade microsatellite instability, whereas Ion Cluster2 had the lowest proportion (Fig. 1C). Prognostic analysis indicated significant differences in overall survival among the four subtypes. Notably, Ion Cluster3 exhibited the longest overall survival and Ion Cluster4 had the poorest prognosis (Fig. 1D).

### Distinctions in biological characteristics and immune infiltration among ion channel subtypes

**Fig. 2:**
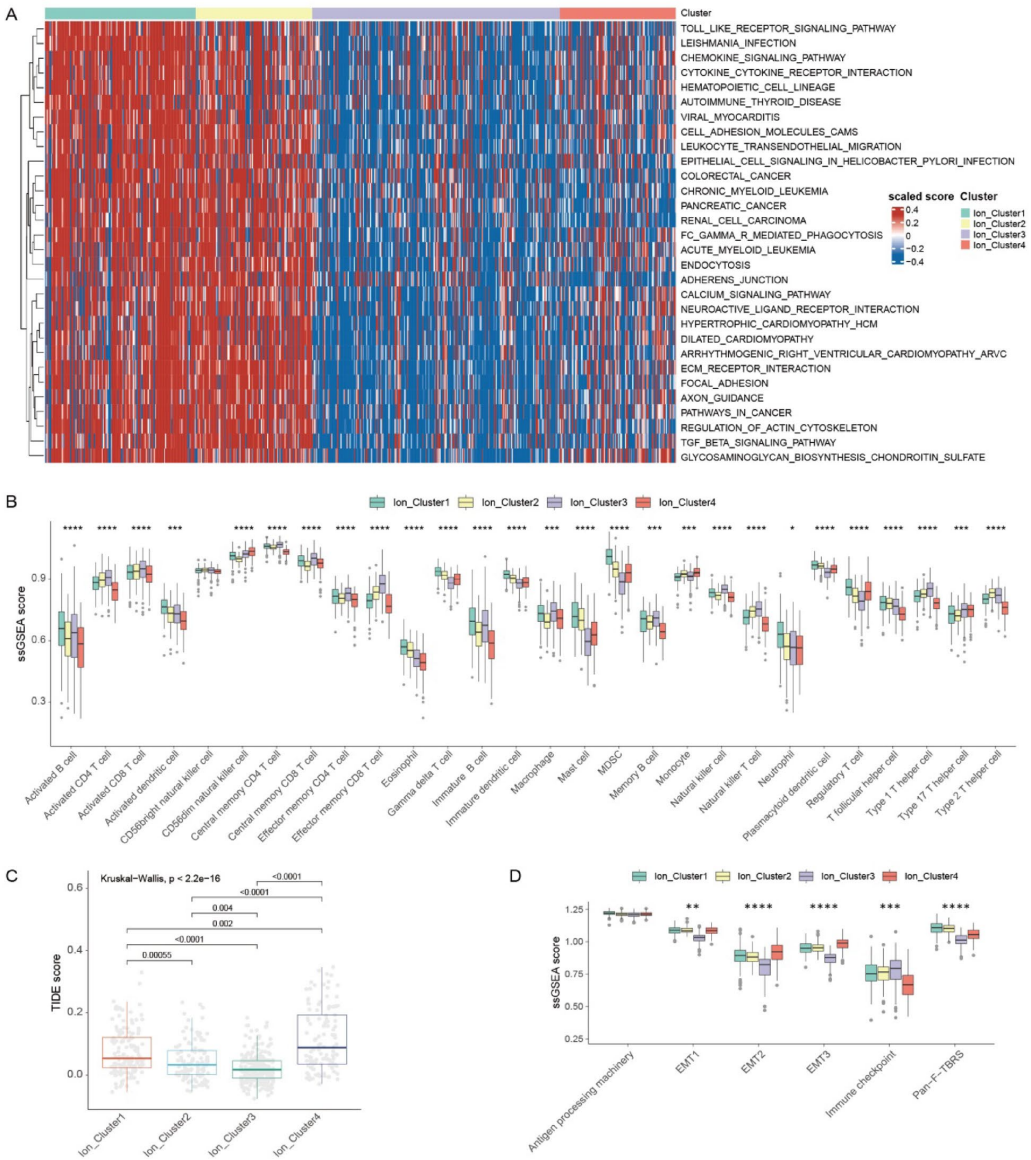
Biological characteristics and immune cell infiltration in four ion channel subtypes. **A** Gene set variation analysis to examine the differences in the biological function of four ion channel subtypes. Blue and red to represent the inhibition and activation of biological pathways respectively. **B** The analysis of Infiltration abundance of 28 immune cells in ion channel subtypes. (Kruskal–Wallis test, *p < 0.05, **p < 0.01, ***p < 0.001, ****p < 0.0001). **C** Differences in the TIDE scores among ion channel subtypes. **D** Differences in epithelial-mesenchymal transition and immune-related pathways among ion channel subtypes. (Kruskal–Wallis test, *p < 0.05, **p < 0.01, ***p < 0.001, ****p < 0.0001). *TIDE* tumor immune dysfunction and exclusion

Pathway analysis was conducted using gene set variation analysis to explore the differences in biological function among the distinct ion channel subtypes. Ion Cluster1 and Ion Cluster2 were significantly enriched in various signaling pathways, including the Toll-like receptor signaling pathway, chemokine signaling pathway, calcium signaling pathway, and transforming growth factor-β signaling pathway (Fig. 2A).

The differences were further investigated in the tumor microenvironment (TME) among the ion channel subtypes. The ssGSEA scores of 28 immune cell types were calculated and compared among the subtypes to assess the immune infiltration levels. There were significant differences in immune infiltration. The level of immune cell infiltration was consistently higher for Ion Cluster3, particularly in activated CD4 and CD8 T cells, while it was relatively lower for Ion Cluster4 (Fig. 2B).

The immune and ESTIMATE scores were also analyzed among the subtypes. Ion Cluster3 exhibited the highest immune and ESTIMATE scores, while Ion Cluster4 was the lowest (Additional file 2: Fig. S3A, B). To validate the immune cell infiltration, the proportions of 22 infiltrating immune cell types in CRC samples were obtained using cell-type identification by estimating relative subsets of RNA transcripts (Additional file 2: Fig. S3C). Consistent with the results of the ssGSEA score, there were similar significant differences in infiltration levels among the subtypes (Additional file 2: Fig. S3D). Besides, TIDE scores were assessed among the ion channel subtypes. Ion Cluster4 obtained the highest score, suggesting the potential limited efficacy of immunotherapy. Ion Cluster3 scored the lowest, indicating a more favorable response to immunotherapy (Fig. 2C). These findings effectively explained the prognosis improvement observed in Cluster3. Significant differences were also found in immune-related and stromal activation pathways among the subtypes (Fig. 2D).

### Integrated analysis of differential expression genes

**Fig. 3:**
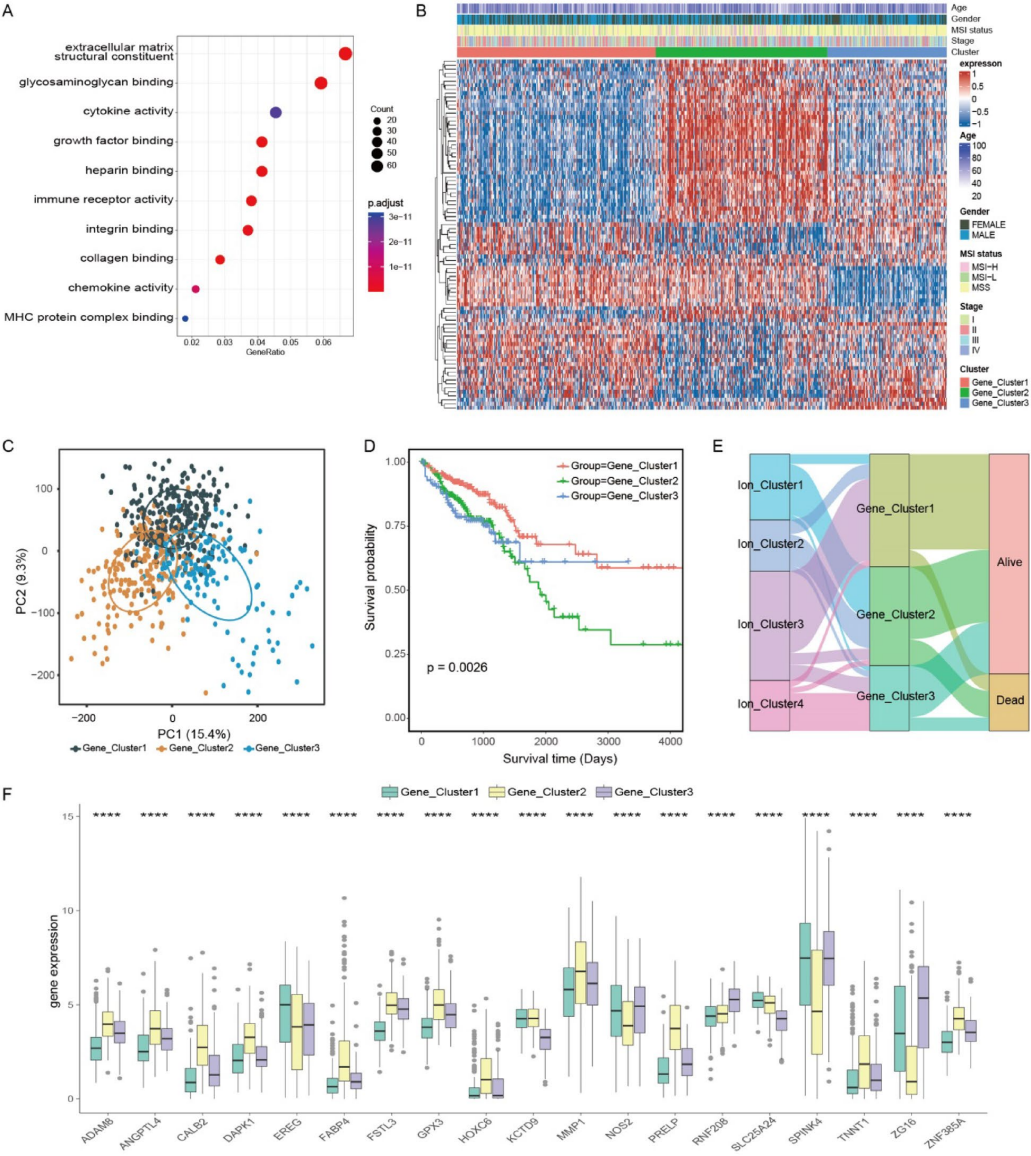
Different expression of ion channel subtypes in colorectal cancer. **A** Gene Ontology enrichment analysis of DEGs was conducted. **B** A Heatmap was used to display the expression of 88 survival-related DEGs in the TCGA CRC dataset. The gene clusters and common clinical variables were used as patient annotations. **C** Three distinct Gene clusters were identified by the principal component analysis of 985 DEGs in the TCGA CRC dataset. **D** Significant differences were shown in the survival curve for gene subtypes (log-rank test, *p* = 0.0026). **E** Alluvial diagram exhibited the association of ion channel subtypes, gene subtypes, and survival states. **F** A total of 19 prognosis-related ion channel DEGs were expressed among the Gene clusters. (Kruskal–Wallis test, *p < 0.05, **p < 0.01, ***p < 0.001, ****p < 0.0001). *DEGs* differentially expressed genes, *TCGA* The Cancer Genome Atlas, *CRC* colorectal cancer

DEGs were analyzed to further investigate the functional differences among distinct ion channel subtypes. As shown in the results, significant differences were observed in the expression of 985 genes across the four subtypes (Additional file 2: Fig. S4A). Enrichment analysis of DEGs using Gene Ontology pathways emphasized their involvement in molecular binding and immune activity (Fig. 3A). Functions such as immune receptor activity and growth factor binding were significantly associated with the DEGs.

A consensus clustering method was performed based on the DEGs. The samples were classified into three gene subtypes (Fig. 3B; Additional file 2: Fig. S4B–D). Principal component analysis indicated no significant differences among the three gene subtypes (Fig. 3C). However, significant differences were found in survival outcomes and the abundance of immune cell infiltration among the gene subtypes. Gene Cluster1 exhibited the most favorable prognosis, with consistently higher levels of immune cell infiltration. In contrast, Gene Cluster2 and Gene Cluster3 showed poorer prognoses and lower levels of immune cell infiltration (Fig. 3D; Additional file 2: Fig. S4E). In the analysis of the predicted response to immunotherapy, the TIDE scores of Gene Cluster3 were notably higher. Such a result indicated a potentially limited efficacy of immunotherapy. The TIDE scores of Gene Cluster1 were the lowest, suggesting a more favorable response to immunotherapy (Additional file 2: Fig. S4F).

The associations were effectively depicted between ion channel subtypes and gene clusters by a Sankey diagram. Gene Cluster1 was primarily originated from Ion Cluster3, while Gene Cluster3 predominantly composed of samples from Ion Cluster4 (Fig. 3E). In addition, there were significant differences in the expression of survival-associated DEGs (*p* < 0.01 in univariate Cox regression) among the gene clusters (Fig. 3F).

### Construction of ion channel gene model

**Fig. 4:**
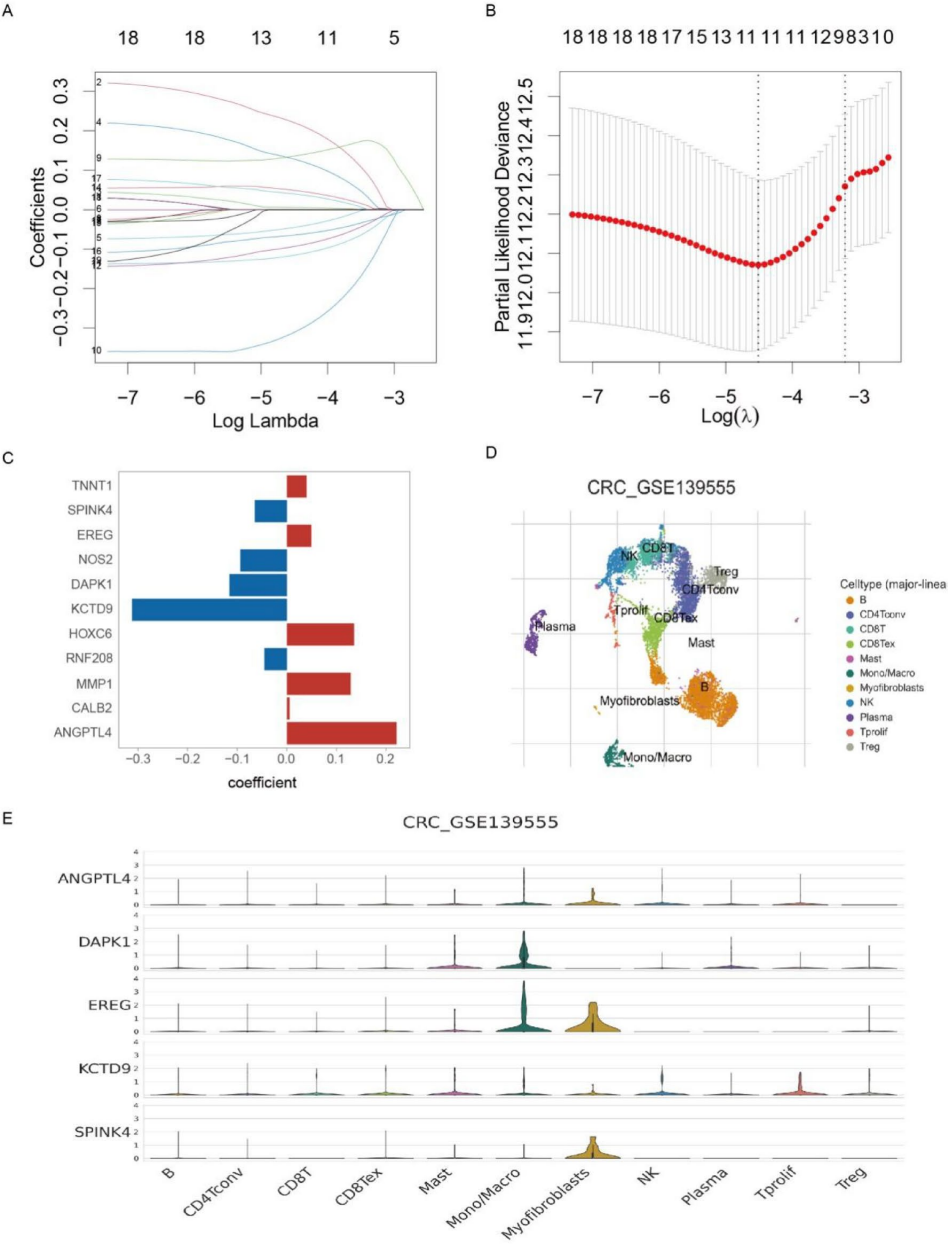
Construction of ion channel models. **A** Relative changes of lambda and variable coefficients in LASSO regression. **B** Cross-validation to identify the corresponding model of lambda.min. **C** Regression coefficients corresponding to the variable genes in the model. **D** Different immune cell subsets in a single-cell cohort [GSE139555]. **E** Expression of ion channel characteristic genes in various cell subsets. *LASSO* the least absolute shrinkage and selection operator regression

A predictive modeling was constructed using the LASSO to explore the clinical implications of ion channel genes and understand their unique characteristics in patients with CRC (Fig. 4A, B). This model was based on 19 DEGs that were strongly associated with survival for each ion channel subtype (*p* < 0.01 in univariate Cox regression, Additional file 2: Fig. S5A). The formula for the model was as follows: Score = (0.2213 × ANGPTL4) + (0.0056 × CALB2) + (0.1286 × MMP1) + (− 0.0453 × RNF208) + (0.1356 × HOXC6) + (− 0.3119 × KCTD9) + (− 0.1158 × DAPK1) + (− 0.0936 × NOS2) + (0.0491 × EREG) + (− 0.0645 × SPINK4) + (0.0400 × TNNT1). Of them, the KCTD9 and ANGPTL4 had the highest weights as protective and risk factors, respectively (Fig. 4C).

**Fig. 5:**
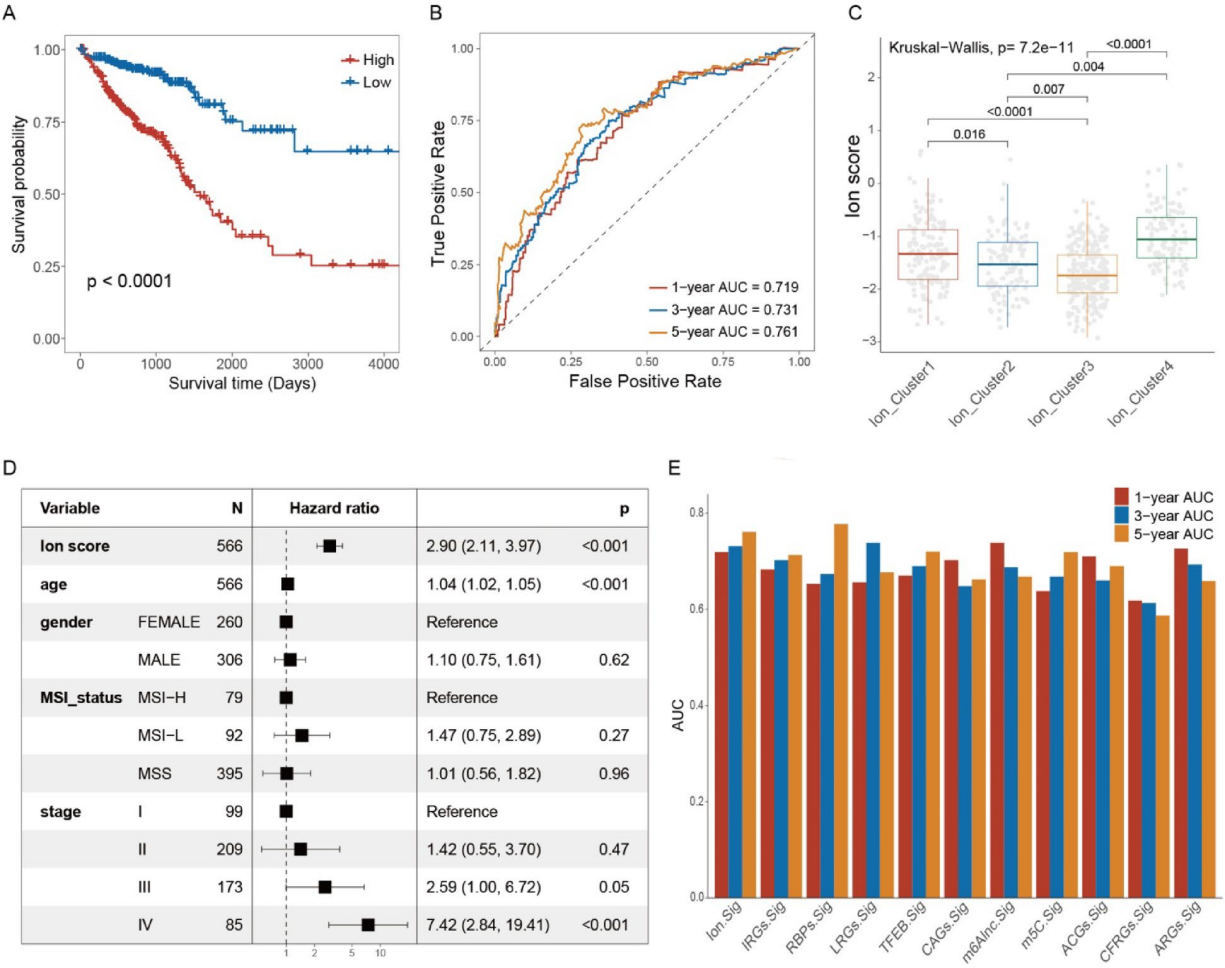
Evaluation of ion channel models. **A** Kaplan–Meier curve showing significant differences between high- and low-score groups in the TCGA (log-rank test, *p* < 0.0001). **B** The predictive ability of ion channel model for the 1-, 3-, and 5-year prognosis of CRC patients in the TCGA. **C** Differences in ion channel score among four subtypes. **D** Multivariate COX regression analysis of ion channel scores. **E** Comparison of the ion channel gene model with published prognostic models for CRC regarding 1, 3, and 5-year survival prediction. Ion.Sig as the model in this study. *TCGA* The Cancer Genome Atlas, *CRC* colorectal cancer

The single-cell dataset GSE139555 was employed to analyze the expression of the model characteristic genes in different immune cell clusters. The results revealed that KCTD9 and ANGPTL4 were expressed in almost all immune cell types (Fig. 4D, E). The formula was applied to generate scores for CRC samples in the TCGA dataset. Unsurprisingly, significant differences in survival rates were discovered between the high and low scores group (Fig. 5A). As indicated by the receiver operating characteristic curve, the ion channel scores were highly strongly predictive of the survival rates at 1, 3, and 5 years (Fig. 5B). Besides, there were significant differences in the ion channel scores among the ion channel subtypes and gene subtypes. The ion channel score of Ion Cluster3 was the lowest among the ion channel subtypes, while Ion Cluster4 was the highest (Fig. 5C). Similarly, Gene Cluster2 demonstrated significantly higher ion channel scores among the gene subtypes, while Gene Cluster1 had the lowest score (Additional file 2: Fig. S5B). These findings were consistent with the earlier observations regarding the prognosis of each subtype.

According to the results of the analysis of univariate Cox regression, the ion channel scores were closely correlated with survival [hazards ratio = 3.43, 95% confidence interval (2.58, 4.55), *p* = 2.09e−17]. Furthermore, the analysis of multivariate Cox regression confirmed a persistent and significant association between ion channel scores and survival (hazards ratio = 2.90, 95% confidence interval [2.11, 3.97], *p* = 3.92e−11; Fig. 5D). We also compiled and compared the area under the curve values of previously published prognostic models for CRC. The ion channel model was at a notably high level in this study (Fig. 5E).

### Performance evaluation of the ion channel predictive modeling

**Fig. 6:**
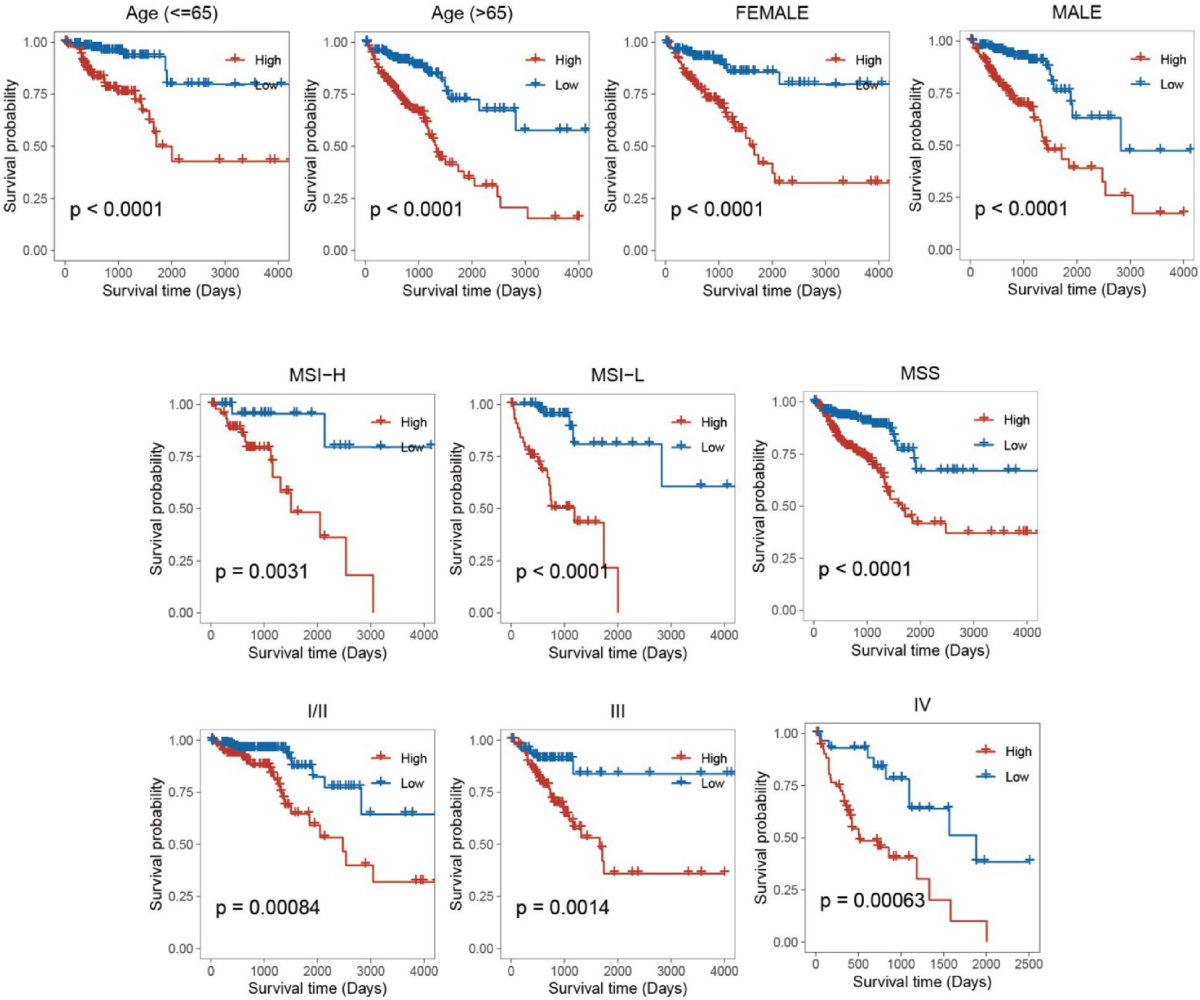
Kaplan–Meier curve showed significant differences between low- and high-score groups under stratification of different clinical variables

**Fig. 7:**
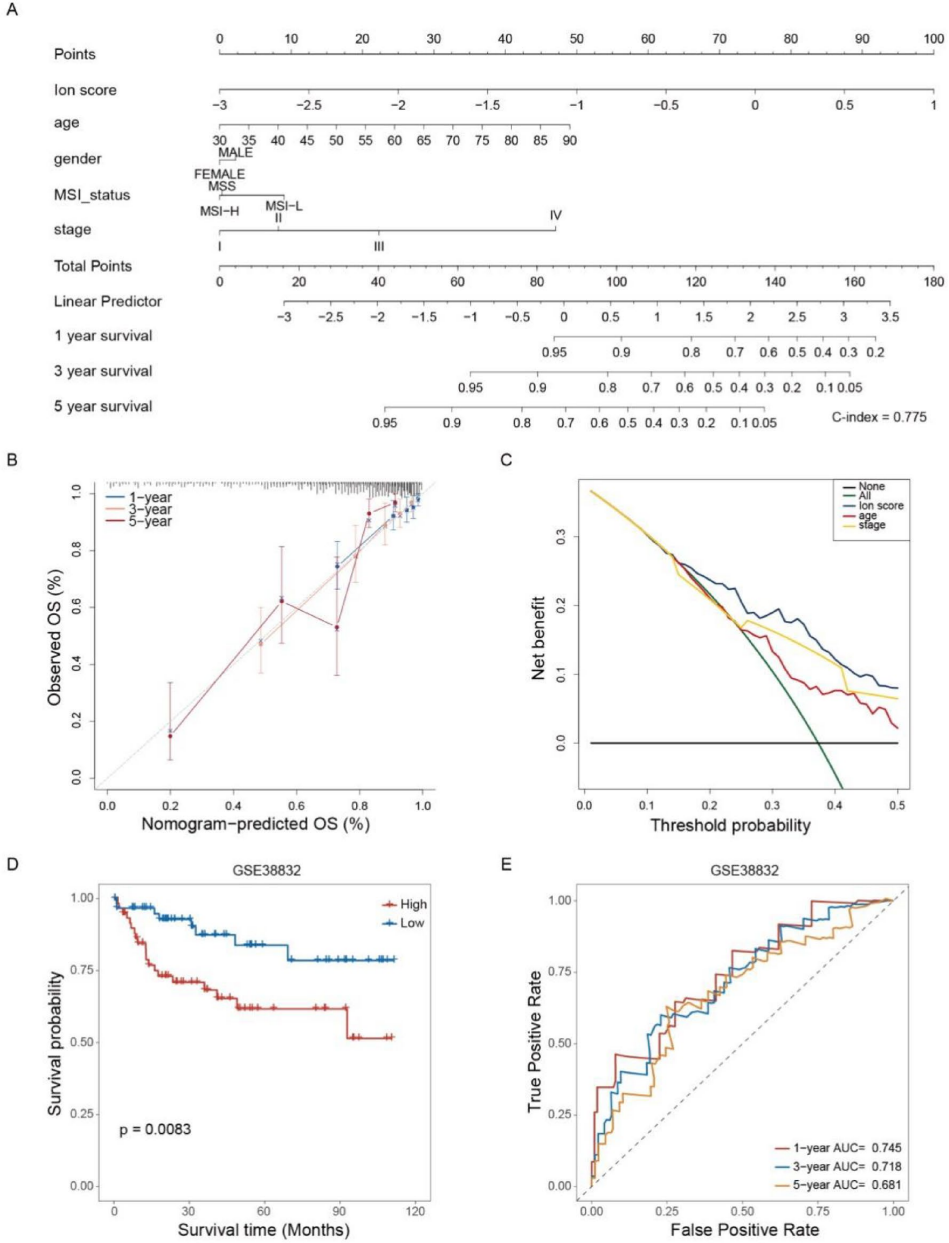
Clinical predictive efficacy of ion channel model. **A** Nomogram for forecasting a patient’s prognosis. **B** Calibration curves for 1-, 3-, and 5-year nomograms. **C** Decision curve analysis for the nomogram. **D** Survival curve showing significant difference between low- and high-score groups in the GSE38832 datasets (log-rank test, *p* = 0.0083). **E** The predictive ability of ion channel model for 1-, 3-, and 5-year prognosis of CRC patients in the GSE38832 datasets

A stratified analysis of clinical variables revealed that the high-score group demonstrated a significantly poorer prognosis across various clinical strata, including age, gender, and tumor stage (Fig. 6). Subsequently, the ability of several clinical variables was assessed, such as age, tumor stage, and model score. Such a process was to predict patient survival in a clinical setting using a nomogram. The ion channel model covered a wider range of scores, reflecting its significant contribution to patient prognosis (Fig. 7A). The calibration curves illustrated the predicted probability of patient survival and the observed probability at 1, 3, and 5 years. The proximity of the two probabilities indicated the strong predictive efficacy of the nomogram (Fig. 7B). Moreover, decision curve analysis showed that the age model, tumor stage model, and ion channel score model had higher net benefits than those of the extreme curves in the significant Pt range of 0.2–0.5. Notably, the clinical value of the ion channel model was the highest (Fig. 7C). Ultimately, the prognostic efficacy of the ion channel model was assessed in the validation set (Fig. 7D, E).

### Features of ion channel model characteristic genes

**Fig. 8:**
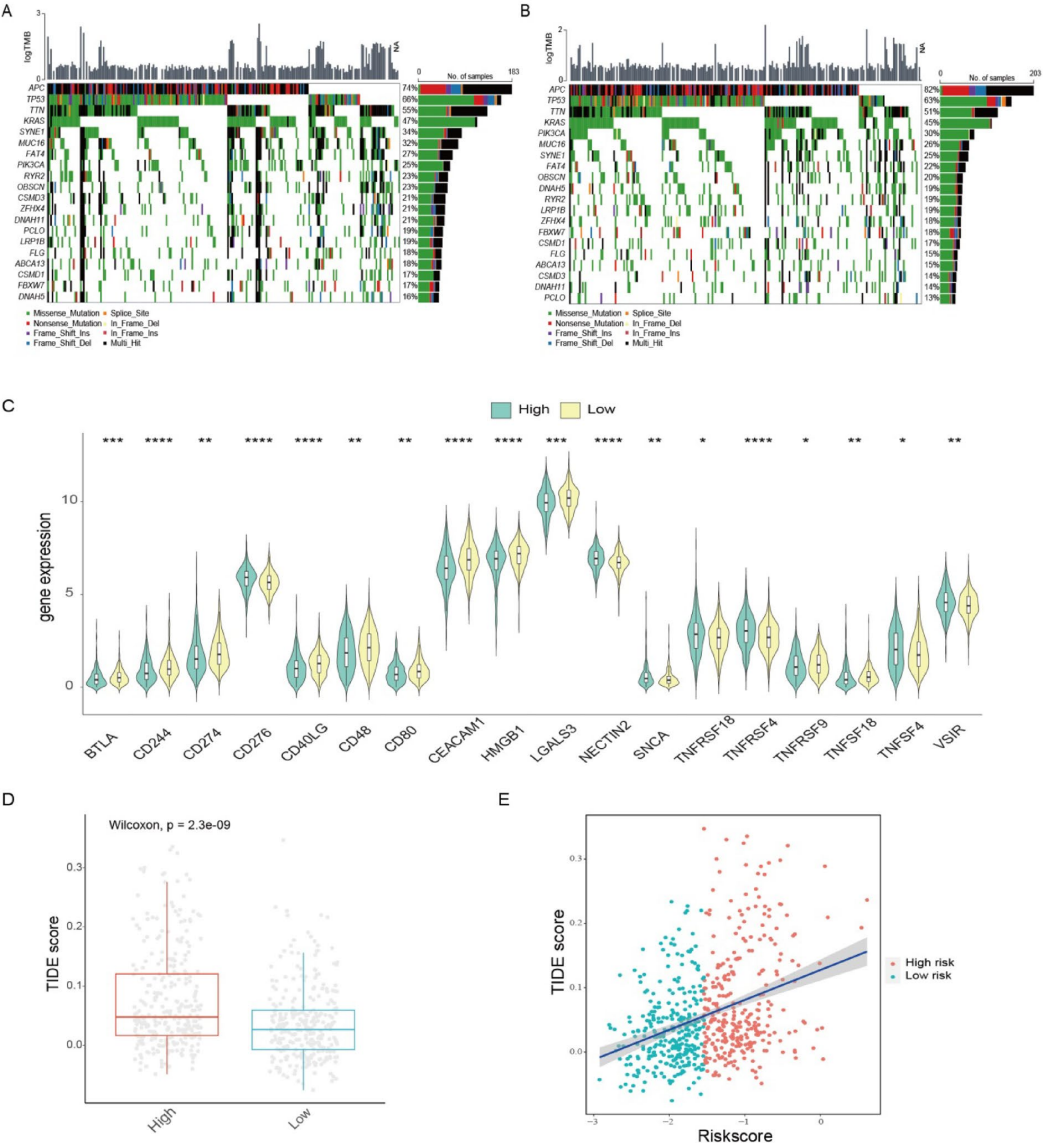
Immunological characteristics and genomic differences between high- and low-score groups. **A**, **B** Waterfall plot to depict the differences in gene mutation landscape between high- (**A**) and low-score (**B**) groups. **C** Different expression of immune checkpoint genes in high- and low-score groups. **D** Differences in the TIDE scores between the low- and high-score groups in the TCGA dataset. **E** Scatterplots to exhibit a positive association between TIDE scores and ion channel scores in the CRC cohort (Spearman Correlation Coefficient; TCGA). *TCGA* The Cancer Genome Atlas, *CRC* colorectal cancer, *TIDE* tumor immune dysfunction and exclusion

There was no significant difference in the mutation burden according to the comparison of the mutation landscape between the high- and low-score groups. However, differences were observed in the mutation frequencies of certain high-frequency genes (Fig. 8A, B). The mutation frequency of SYNE1 was markedly higher in the high-score group (*p* = 0.023), and the mutation frequency of APC represented a remarkable uptrend in the low-score group (*p* = 0.038).

**Fig. 9:**
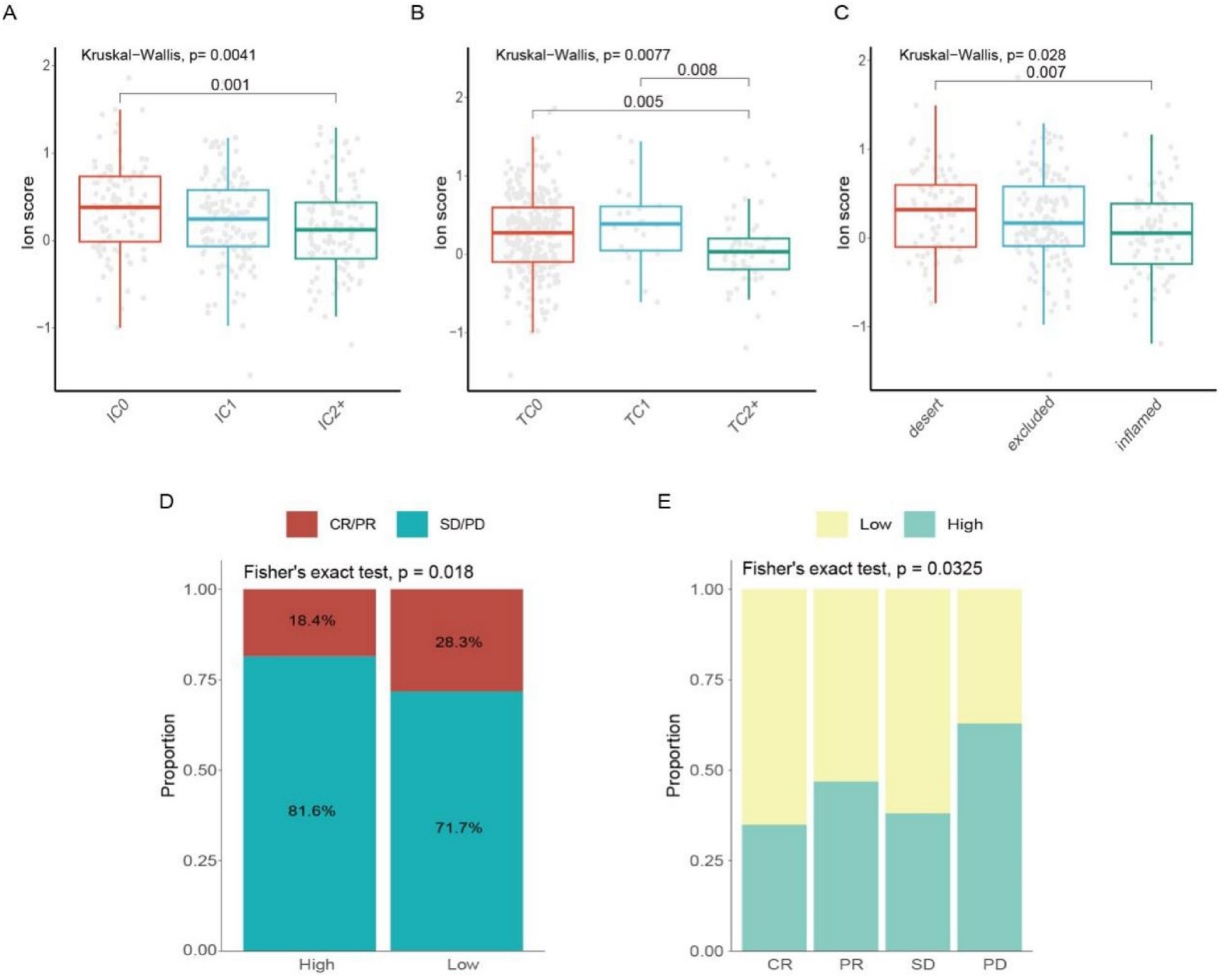
Analysis of the IMvigor210 immunotherapy cohort. **A**, **B** Changes of ion channel scores among various IC groups/TC groups. **C** Changes in ion channel score among different immune subtypes. **D** Comparison of the immunotherapy responses (CR, PR, SD, PD) between high- and low-score groups. **E** The percentage of patients in each anti-PD-L1 response group with low and high ion channel scores. *IC* tumor-infiltrating immune cells, *TC* tumor cells, *CR* complete response, *PR* partial response, *SD* stable disease, *PD* progressive disease

We compared immune infiltration and the expression of immune checkpoint markers between the two groups. The high-score group primarily exhibited infiltration of CD56dim natural killer cells and up-regulation of genes such as CD276, TNFRSF4, and VSIR. In contrast, the low-score group displayed infiltration of activated B cells, activated CD4 and CD8 T cells, with higher expression of genes such as CD244, CD274, and CD48 (Fig. 8C; Additional file 2: Fig. S5D). Based on a comparison of TIDE scores between the two groups, the TIDE scores were notably raised in the high-score group, suggesting that the response to immunotherapy was weakened (Fig. 8D, E). The ion channel scoring system was used to analyze the IMvigor210 cohort to evaluate the correlation between ion channel scores and the response to immunotherapy. The ion channel scores were significantly associated with the expression levels of programmed cell death ligand-1 (PD-L1) in immune cell/tumor cell (IC/TC) groups. The ion channel scores were considerably lower in the high PD-L1 (IC2+ and TC2+) expression groups (Fig. 9A, B). Furthermore, there was an increase in scores in the desert subtype, while scores in the inflamed subtype significantly decreased (Fig. 9C). The analysis of immunotherapy outcome demonstrated a higher complete response/partial response rate in the low-score group (Fig. 9D, E). Ultimately, the prognostic outcomes of the two groups were compared in the cohort. Although the differences were no statistical significance, the survival outcomes tended to be poorer in the high-score group (Additional file 2: Fig. S5C). This absence of statistical significance might be attributed to the differences in cancer types.

Overall, these findings provided compelling evidence that the ion channel scoring system held promise as a valuable tool for predicting treatment efficacy and guiding immunotherapy interventions.

### Analysis of drug sensitivity prediction

**Fig. 10:**
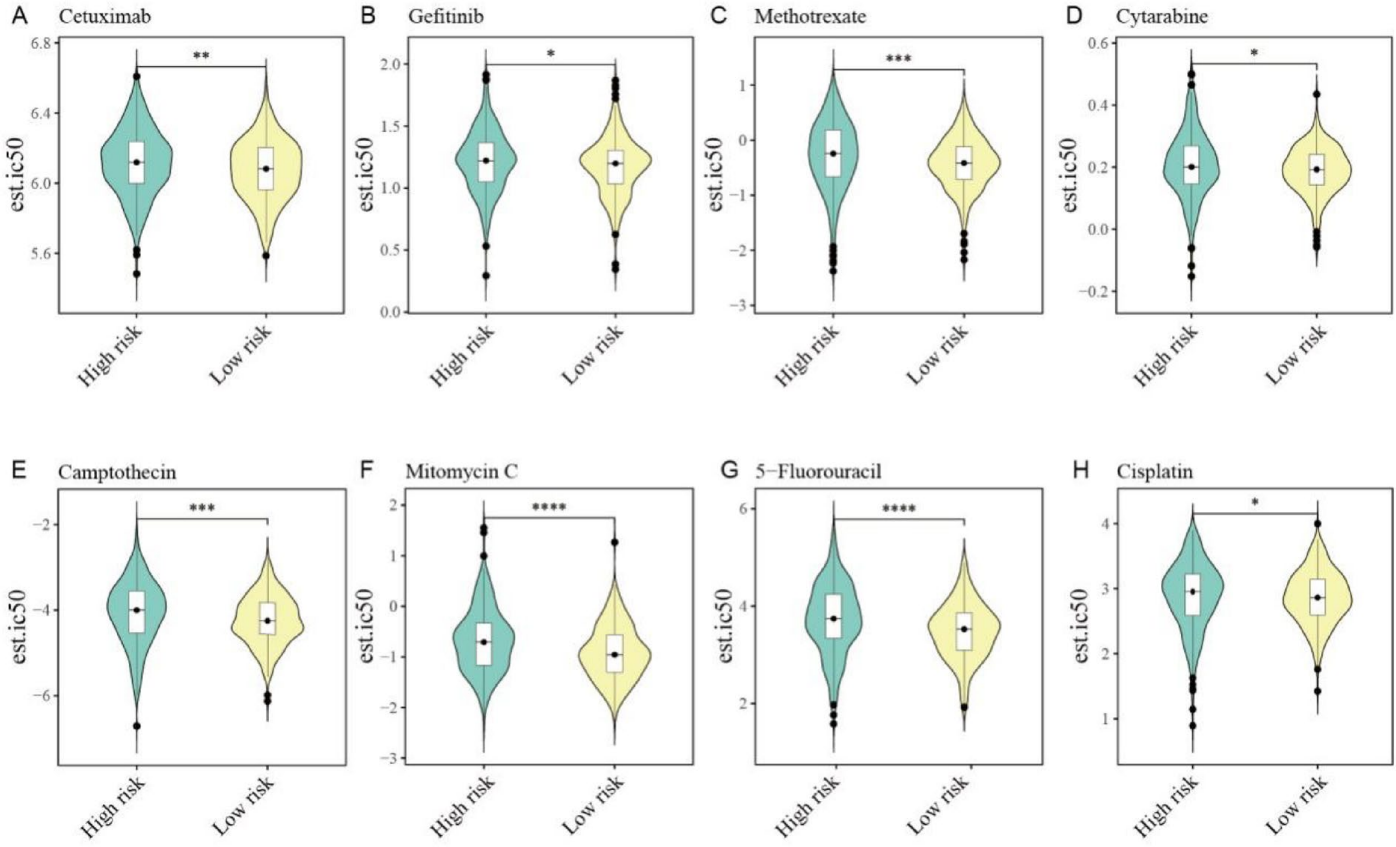
Drug sensitivity analysis. **A**–**H** Violin plots of the predicted IC50 for **A** cetuximab, **B** gefitinib, **C** methotrexate, **D** cytarabine, **E** camptothecin, **F** mitomycin C, **G** 5-fluorouracil, **H** Cisplatin between the groups with a low and high score in the TCGA dataset. *IC50* half-maximal inhibitory concentration

In the drug sensitivity analysis, the pRRophetic function was applied to predict the IC50 of different drugs in the high- and low-score groups. We analyzed various drugs, including first-line therapies such as cetuximab, 5-fluorouracil, and cisplatin, as well as other commonly used anti-cancer drugs including methotrexate, mitomycin C, and gefitinib. The IC50 consistently remained lower in the low-score group, indicating higher drug sensitivity (Fig. 10A–H). The above results confirmed the predictive capability of the ion channel scoring system in assessing drug efficacy and highlighted the important role of ion channels as indicators for drug response.

### Experimental validation of core genes

**Fig. 11:**
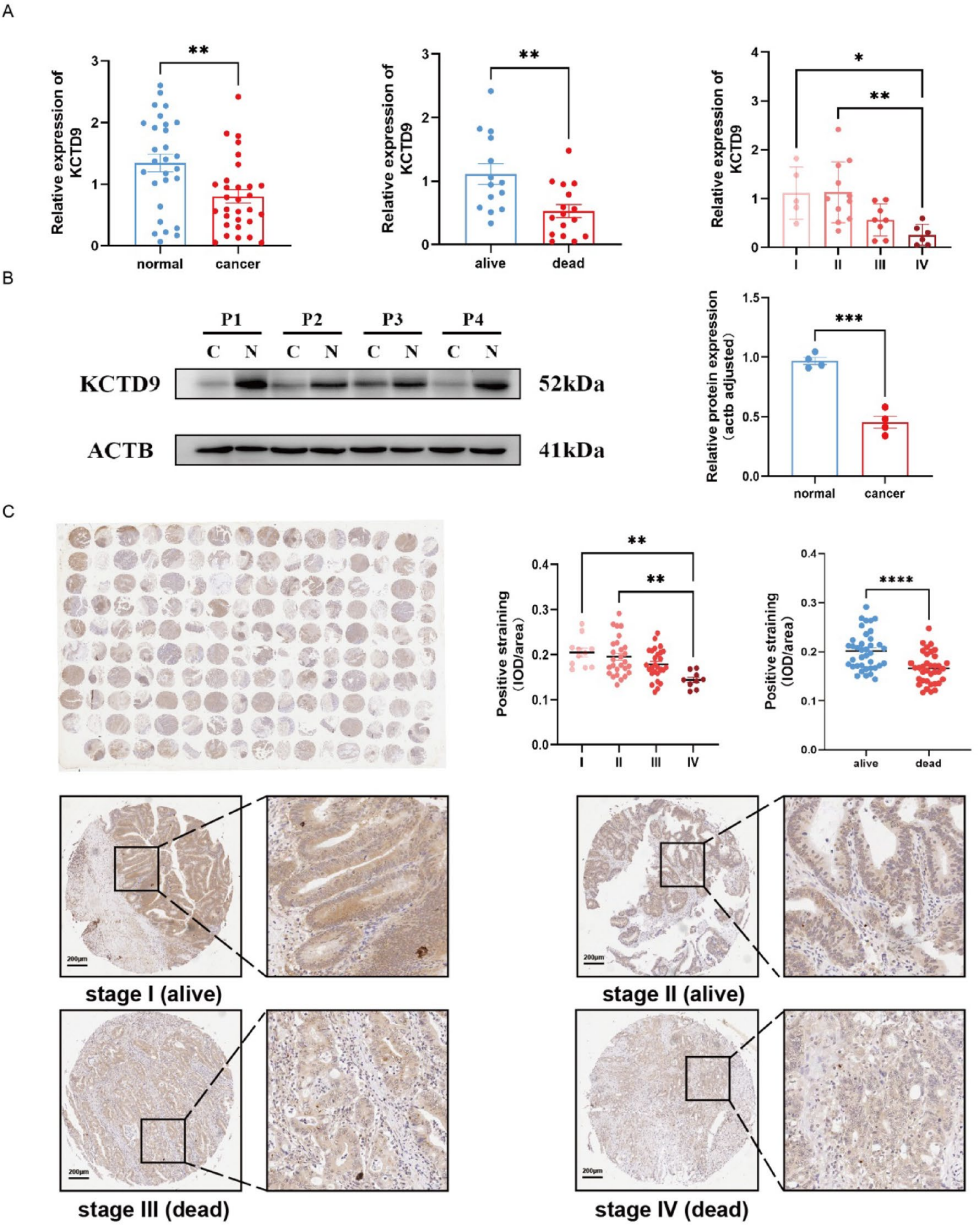
Experimental validation of characteristic gene expression in colorectal cancer. **A** Detection of KCTD9 mRNA expression patterns in CRC and paracancerous tissue by RT-qPCR. **B**, **C** Detection of KCTD9 protein expression patterns in CRC tissues and paracancerous tissue samples by the western blot (**B**) and the immunohistochemistry (**C**) in the tissue microarray. *IOD* Integral optical density, *I–IV* TNM staging of patients, *alive/dead* survival information of the patient in the 5th year after surgery, *KCTD9* potassium channel tetramerization domain containing 9, *CRC* colorectal cancer, *RT-qPCR* real-time quantitative polymerase chain reaction

Molecular experiments focusing on KCTD9 were conducted to assess the clinical value of the core genes. A CRC cDNA array containing prognostic information was subjected to RT-qPCR analysis to examine the mRNA expression pattern of KCTD9 in CRC and adjacent tissues (Fig. 11A). The protein-level expression of KCTD9 was evaluated by western blot and immunohistochemical staining (Fig. 11B, C). The results showed that KCTD9 was expressed in both CRC and adjacent tissues. The expression of KCTD9 was significantly down-regulated in CRC, particularly at higher TNM stages, which was often associated with unfavorable prognosis. Such a result was consistent with the outcome of bioinformatics analysis.

### KCTD9 inhibits the proliferation, migration and invasion capability of LOVO cells

**Fig. 12:**
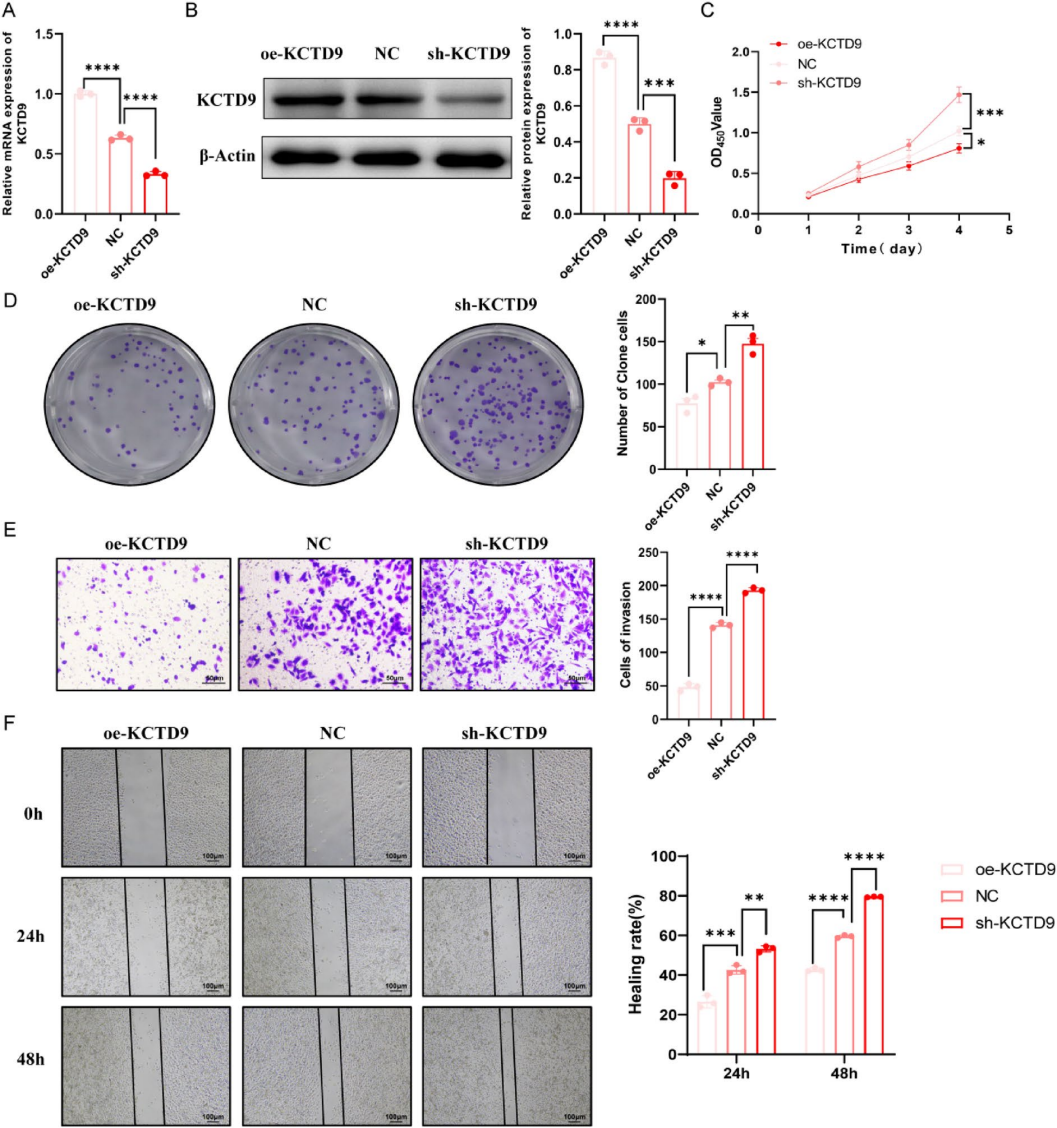
KCTD9 inhibited the proliferation, migration and invasion capability of LOVO cells. **A** Detection of mRNA expression patterns of KCTD9 in LOVO cells by RT-qPCR. **B** Detection of KCTD9 protein expression in LOVO cells by western blot. **C** CCK-8 assay to detect the cell viability of LOVO cells. **D** Colony formation assay to measure the proliferation capability of LOVO cells. **E** Transwell invasion assay to determine the invasion capability of LOVO cells. **F** Wound healing assay to evaluate the migration capability of LOVO cells. *p < 0.05, **p < 0.01, ***p < 0.001, ****p < 0.0001. *KCTD9* potassium channel tetramerization domain containing 9, *RT-qPCR* real-time quantitative polymerase chain reaction

We modulated the KCTD9 expression in LOVO cells and conducted a series of cellular functional experiments to further explore the effect of KCTD9 in the malignant phenotype of colon cancer. The results of RT-qPCR and western blot revealed that compared with the NC group, the expression of KCTD9 was significantly increased in the oe-KCTD9 group, while the expression of KCTD9 was notably decreased in the sh-KCTD9 group (*p* < 0.0001, Fig. 12A, B). Subsequently, the CCK-8 results displayed that in contrast to the NC cells, a markedly retarded growth rate was observed in oe-KCTD9 cells, while the proliferation capability was elevated in sh-KCTD9 cells (*p* < 0.01 or *p* < 0.001) (Fig. 12C). These findings were confirmed by the colony formation assay (Fig. 12D). Besides, the invasive potential assessed in Fig. 12E was consistent with these observations, with diminished invasion in oe-KCTD9 cells and a significantly enhanced invasion in sh-KCTD9 cells (*p* < 0.0001). Ultimately, a different migratory response was quantitatively illustrated by the wound healing assay in Fig. 12F; and the healing rate was reduced in oe-KCTD9 cells, while sh-KCTD9 cells exhibited a faster closure rate at both 24 and 48 h post-wounding (*p* < 0.01 or *p* < 0.0001). In summary, these results suggested that KCTD9 played a significant role in LOVO cell proliferation, migration and invasion.

## Discussion

CRC poses a major global public health challenge due to its high incidence, mortality rate, and increasing prevalence among the younger population [1, 2]. Such disease is known for its heterogeneity, leading to complexity in tumor characteristics suboptimal outcomes [5]. Hence, accurate molecular subtypes of CRC are crucial for guiding personalized therapies [6]. In 2015, the CRC Subtyping Consortium classified CRC into four consensus molecular subtypes (CMS1–4), marking a significant advancement [39]. Besides, subsequent studies proposed additional molecular subtypes [40–42]. However, there is still significant heterogeneity within each subtype. Therefore, it is crucial to develop more accurate classification schemes and predictive modelings. These advancements will contribute to the use of personalized treatment approaches based on accurate classifiers and predictive modelings, ultimately improving patient survival rates.

Ion channels, transmembrane proteins responsible for selective ion transport, play a critical role in regulating tumor cell behavior [43–45] and the TME [46]. Their widespread presence in cells makes them valuable indicators for cancer diagnosis, therapeutic targeting, and prognosis [47]. Therefore, the classification of CRC patients into subtypes based on ion channel gene expression holds significant study value, which was demonstrated by the results of this study.

In this study, TCGA CRC samples were classified into four subtypes (Ion Cluster1–4) based on the expression of 279 ion channel genes using a consensus clustering method. The ion channel subtypes were significantly correlated with microsatellite instability and prognosis. This study further explored the interrelation between ion channel subtypes and cellular infiltration in the TME based on previous studies on the association between ion channels and tumor immunity [48, 49], as well as the influence of microsatellite stability on immunotherapy efficacy.

Immune cell infiltration significantly varied among the subtypes. Ion Cluster3 is associated with the most favorable prognosis, characterized by a significant increase in infiltration of activated CD4 and CD8 T cells. In contrast, Ion Cluster4, associated with the poorest prognosis, exhibited a lower level of immune cell infiltration. CD8+ T cells have been reported to possess cytotoxic functions [50, 51], while CD4+ T cells play a supportive role [52, 53] and directly counteract tumors [54, 55]. Macrophages exhibit a sophisticated function in tumor progression [56]. Neutrophils [57] and B cells [58] also play important roles in the TME. Neutrophils can secrete cytokines to promote cancer progression, while dense infiltration of B cells indicates a favorable tumor prognosis. The immune infiltration results of this study are generally consistent with these published conclusions. To gain deeper insights into the functional disparities among ion channel subtypes, we performed Gene Ontology enrichment analysis on DEGs associated with these subtypes. The DEGs were found to be involved in molecular binding and immune activity. The samples were reclassified into three gene subtypes based on DEGs, and a close correspondence between gene subtypes and ion channel subtypes was observed, as depicted in the Sankey diagram. The survival outcomes and immune cell infiltration among gene subtypes verified the differences observed among ion channel subtypes. Such a result supported the reliability of the ion channel subtype classification. These findings deepen the understanding of the interaction among ion channels, immune cell infiltration, and CRC prognosis.

Given the role of ion channels in CRC heterogeneity, immune cell infiltration, and prognosis, a predictive modeling was constructed using LASSO regression to quantitatively capture the ion channel properties. Consistent with published studies, 11 ion channel genes in the model were found to be associated with tumor prognosis. Notably, the protective factors included KCTD9 [59], DAPK1 [60], NOS2 [61], and SPINK4 [62], and the risk factors for poor CRC prognosis were listed as follows: ANGPTL4 [63], MMP1 [64], HOXC6 [65], EREG [66], TNNT1 [67], and CALB2. The exact role of RNF208 in CRC is not yet fully understood, but evidence suggests that its overexpression can inhibit tumor formation and lung metastasis of triple-negative breast cancer cells [68]. Such evidence can justify its inclusion in the model. These findings demonstrate the potential of the ion channel score as an independent prognostic biomarker for patients with CRC. As expected, the score of Ion Cluster4 with a poor prognosis was the highest, while Ion Cluster3 with a favorable prognosis had the lowest score.

To understand the characteristics of ion channel scoring, we analyzed the differences in mutational landscape between the groups with high- and low-score groups. The results revealed numerous gene mutations, with significant differences in the mutation frequencies of APC and SYNE1. As displayed in a previous study, APC mutation was related to immune evasion in CRC [69], which was consistent with the favorable immunotherapy response in the low-score group. Furthermore, SYNE1 mutation has been linked to shorter overall survival in CRC patients with liver metastasis [70], and APC mutation exhibited longer overall survival [71]. These could be reasonable explanations for the poorer prognosis in the high-score group and the better prognosis in the low-score group. In addition, we compared immune cell infiltration and the expression of immune checkpoint molecules between the high- and low-score groups, providing insights into the potential significance of the ion channel score in predicting the efficacy of immunotherapy. Ultimately, the findings in this study were validated in the IMvigor210 immunotherapy cohort. The survival rate was lower in the high-score group of this cohort. The expression level of PD-L1 was negatively correlated with ion channel scores. In addition, patients with low ion channel scores exhibited better immunotherapy than those with high scores. Overall, the predictive modeling served as a valuable tool for assessing tumor immune status and guiding immunotherapy strategies.

Another significant finding in this study was the correlation between the ion channel score and sensitivity to chemotherapy and targeted drugs. Significant differences were observed in drug sensitivity between the low- and high-score groups. Subgroups with lower scores tended to be more responsive to drugs. Therefore, the model score system could guide the prescription of individualized clinical drugs and the development of new pharmaceuticals.

To confirm the clinical relevance of bioinformatics analysis, we conducted experimental validations. Notably, KCTD9, which had the highest absolute coefficient in the prognostic model, significantly influenced the scoring outcomes. Single-cell analysis showed that KCTD9 was expressed in all immune cell types, distinguishing it from the other 10 characteristic genes. There is a significant difference in the expression of KCTD9 between cancerous and adjacent tissues. Given the above reasons and the limited studies on the relationship between KCTD9 and clinical features of CRC, we specifically focused on validating its role in this study. KCTD9 was observed to act as a tumor suppressor by exhibiting low expression levels in CRC tissues. Moreover, its expression was higher in the early stages than in the intermediate and advanced stages of CRC. Importantly, the down-regulation of KCTD9 was often correlated with unfavorable prognosis in CRC. These experimental findings were consistent with the bioinformatics analysis, reflecting the practicality and reliability of the predictive modeling to some extent.

In addition, the observed regulatory effect of KCTD9 expression in LOVO cells highlighted the critical role of this gene in regulating oncogenic behavior. Overexpression of KCTD9 can inhibit cell proliferation, and reduce invasion and migration capabilities, suggesting a suppressive function in tumor progression. These results were in line with prior studies that KCTD9 is a potential tumor suppressor in CRC [72]. In contrast, the knockdown of KCTD9 resulted in enhanced proliferation and invasion, supporting the notion that reduced expression of this gene could contribute to an invasive cancer phenotype. Notably, these findings aligned with the emerging understanding of the complex role played by potassium ion channel tetramerization domain-containing proteins in cancer biology [73]. The distinct behaviors observed upon KCTD9 manipulation might offer insights into therapeutic strategies that could restore or inhibit its function, potentially curbing the invasion of CRC. Further studies were needed to elucidate the mechanisms underlying these observations and to explore the potential of KCTD9 as a biomarker for cancer progression and prognosis.

There are a few limitations in this study. First, despite some experimental validation, it heavily relies on public databases. Such a limitation underscores the need for additional in vivo and in vitro studies to support the findings in this study. Second, this analysis only focused on ion channel genes, potentially overlooking other critical pathways in CRC. Nevertheless, the results obtained from this study remain highly practical.

Overall, CRC was classified into four molecular subtypes in this study based on ion channel genes and a predictive modeling was developed. Such classification and the model scores provide valuable information regarding CRC prognosis, immune cell infiltration patterns within the TME, response to immunotherapy, and drug sensitivity. Besides, the cellular functional experiments confirm the tumor-suppressive effect of the core gene KCTD9. These findings are of significant importance for predicting prognosis and guiding personalized treatment strategies for patients with CRC.

### Supplementary Information

**Additional file 1: Table S1.** Primer sequence of KCTD9.

**Additional file 2: Figure S1.** Flowchart of the study. **Figure S2.** (A) Forest plot showing 36 ion channel genes significantly associated with prognosis identified by univariate cox regression. (B–D) Indicators of different number of clusters in the consistency clustering method. **Figure S3.** (A, B) Box plots demonstrating the variations in immunization scores and ESTIMATE scores among ion channel subtypes. (C) The proportion of 22 immune cell subsets in 619 CRC samples. (D) Box plot showing the differences in the proportion of 22 immune cell infiltration among ion channel subtypes. **Figure S4.** (A) Venn Diagram showing the amount of intersections of differentially expressed genes among each ion channel subtype. (B–D) Indicators of different number of clusters in the consistency clustering based on DEGs. (E) 28 immune cells infiltration abundance of three gene subtypes. (F) Differences in the TIDE scores of gene subtypes. **Figure S5.** (A) Forest plot demonstrating prognosis-related DEGs among ion channel subtypes. (B) Kaplan–Meier curve showing differences between low- and high-scoring groups in the IMvigor210 cohort. (C) Difference of ion channel score among three Gene clusters in the TCGA. (D) 28 immune cells infiltration abundance in the high- and low-scoring groups.

## Data Availability

The experimental data that support the findings of this study are openly available on the figshare website (10.6084/m9.figshare). The bioinformatic data analyzed during the current study are available on the figshare website (10.6084/m9.figshare). These data were derived from the following public domain resource: Genome Data Commons (https://www.cancer.gov/ccg/research/genome-sequencing/tcga). Genome Data Commons (https://gdc.cancer.gov/about-data/publications/pancanatlas). TIMER (http://timer.cistrome.org/). MSigDB (http://www.gsea-msigdb.org/gsea/msigdb/human/collections.jsp). TIDE (http://tide.dfci.harvard.edu/). Imvigor210 (http://research-pub.gene.com/IMvigor210CoreBiologies/). TISCH (http://tisch.comp-genomics.org/home/). GEO (http://www.ncbi.nlm.nih.gov/geo). HUGO Gene Nomenclature Committee (https://www.genenames.org/).
