# Supplementary material for: Molecular subtyping and the construction of a predictive model of colorectal cancer based on ion channel genes
Source: Eur J Med Res. 2024 Apr 4;29:219. doi: 10.1186/s40001-024-01819-2 (PMC10993535; doi:10.1186/s40001-024-01819-2)
Supplement: Supplementary file 1 — Additional file 1: Table S1. Primer sequence of KCTD9. [file 40001_2024_1819_MOESM1_ESM.docx]

**Table S1.** Primer sequence of KCTD9

| **Primer Information** | |  |
| --- | --- | --- |
| Gene of interest | Amplified fragment length (bp) | |
| KCTD9 | 242 |  |
| Forward primer | Reverse primer |  |
| ACCTCCCTACCAATGACT | ATCTCCTCCCACTATGC |  |
