# Supplementary material for: Molecular subtyping and the construction of a predictive model of colorectal cancer based on ion channel genes
Source: Eur J Med Res. 2024 Apr 4;29:219. doi: 10.1186/s40001-024-01819-2 (PMC10993535; doi:10.1186/s40001-024-01819-2)

**Figure S1.** Flowchart of the study

**
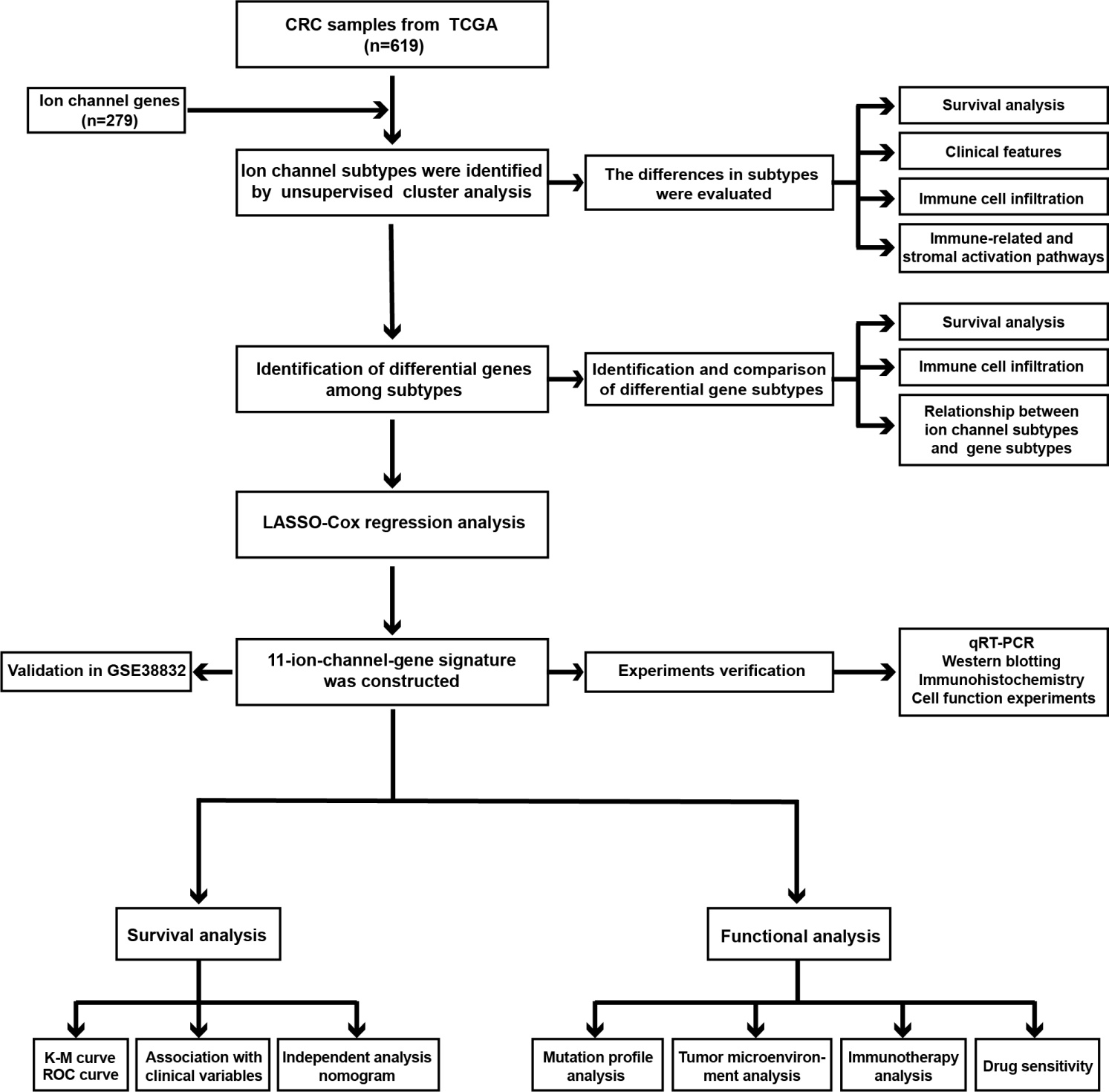
**

**Figure S2.** (A) Forest plot showing 36 ion channel genes significantly associated with prognosis identified by univariate cox regression. (B-D) Indicators of different number of clusters in the consistency clustering method.


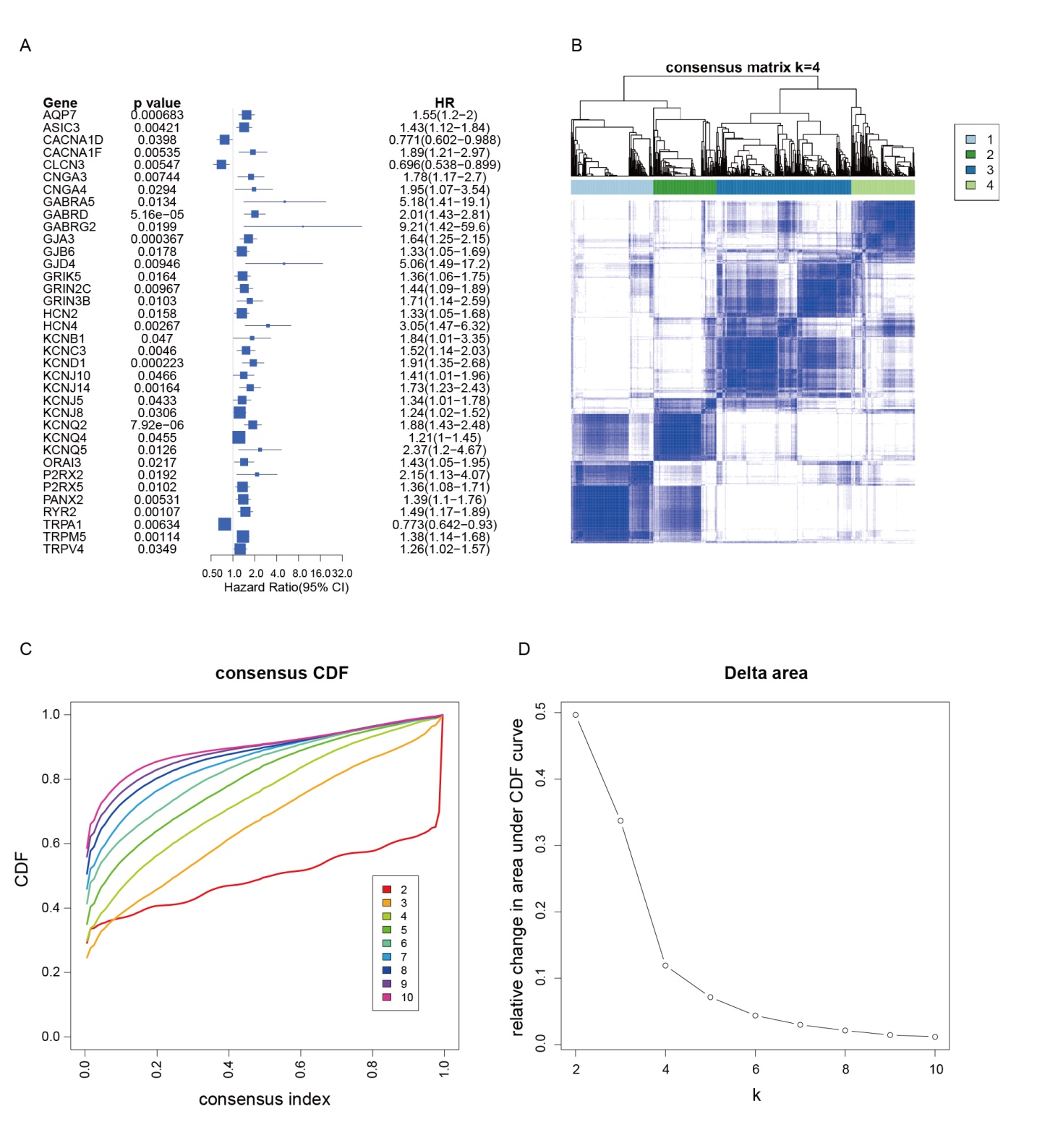


**Figure S3.** (A-B) Box plots demonstrating the variations in immunization scores and ESTIMATE scores among ion channel subtypes. (C) The proportion of 22 immune cell subsets in 619 CRC samples. (D) Box plot showing the differences in the proportion of 22 immune cell infiltration among ion channel subtypes.


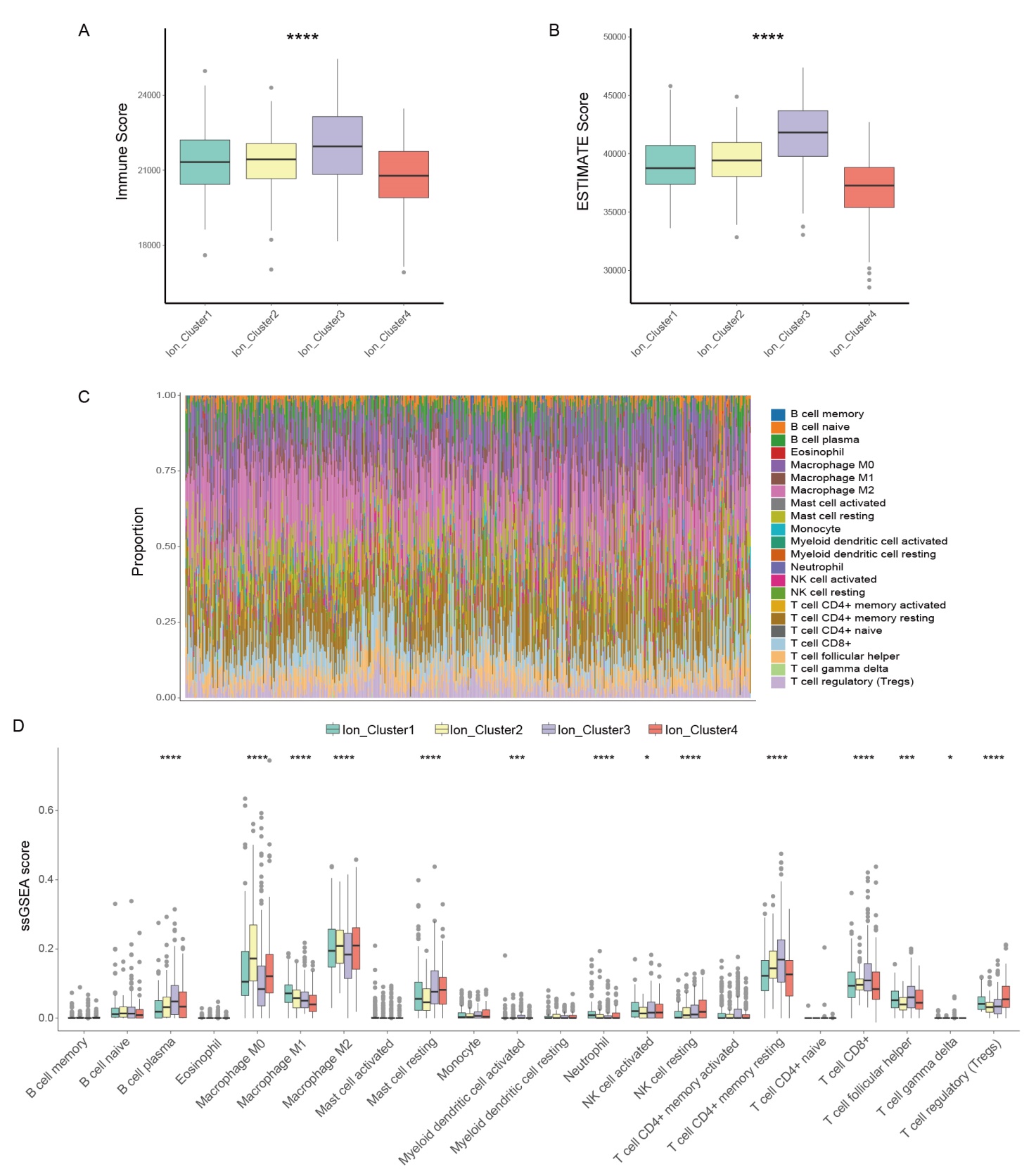


**Figure S4.** (A) Venn Diagram showing the amount of intersections of differentially expressed genes among each ion channel subtype. (B-D) Indicators of different number of clusters in the consistency clustering based on DEGs. (E) 28 immune cells infiltration abundance of three gene subtypes. (F) Differences in the TIDE scores of gene subtypes.


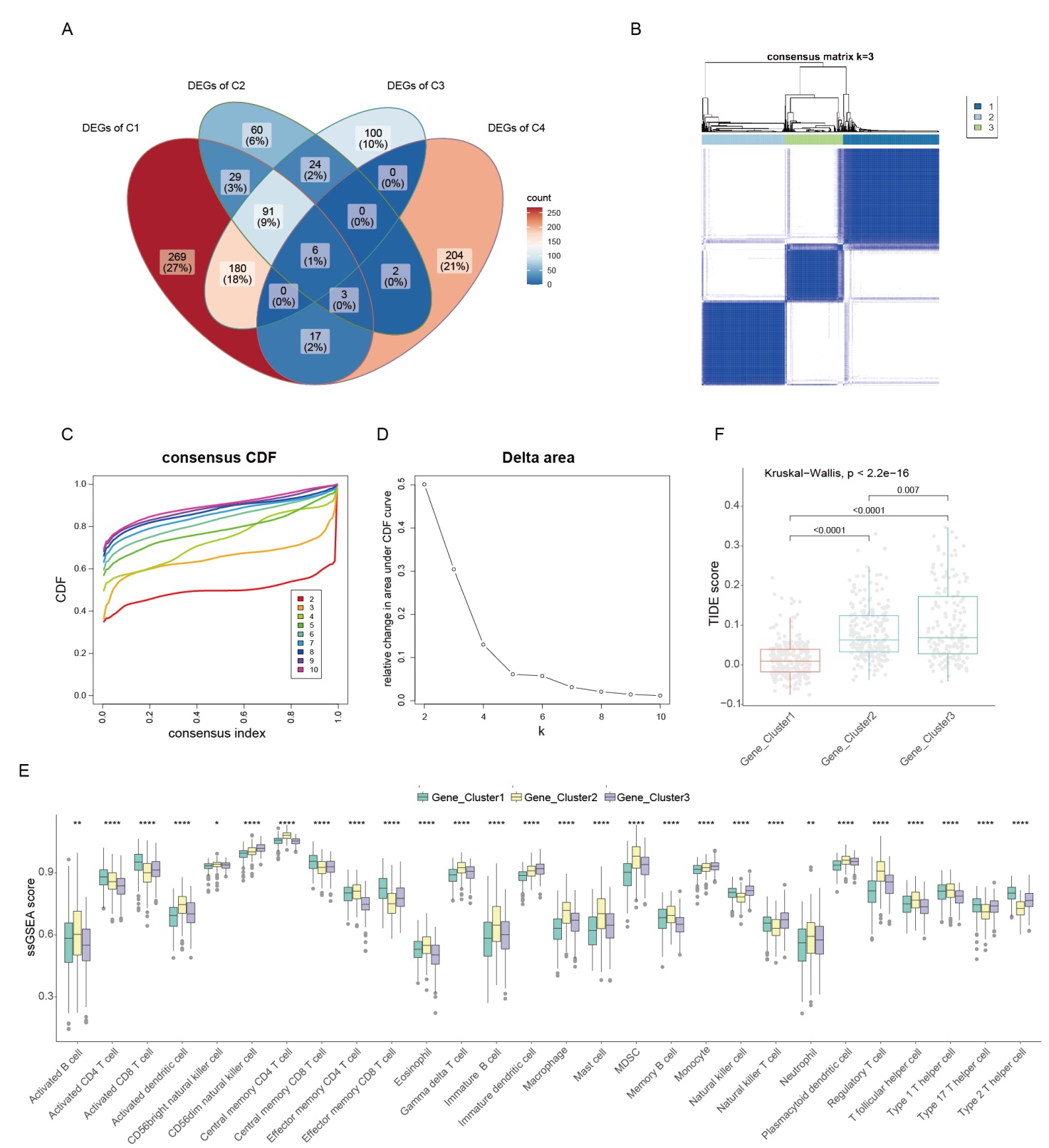


**Figure S5.** (A) Forest plot demonstrating prognosis-related DEGs among ion channel subtypes. (B) Kaplan-Meier curve showing differences between low- and high-scoring groups in the IMvigor210 cohort. (C) Difference of ion channel score among three Gene clusters in the TCGA. (D) 28 immune cells infiltration abundance in the high- and low-scoring groups.


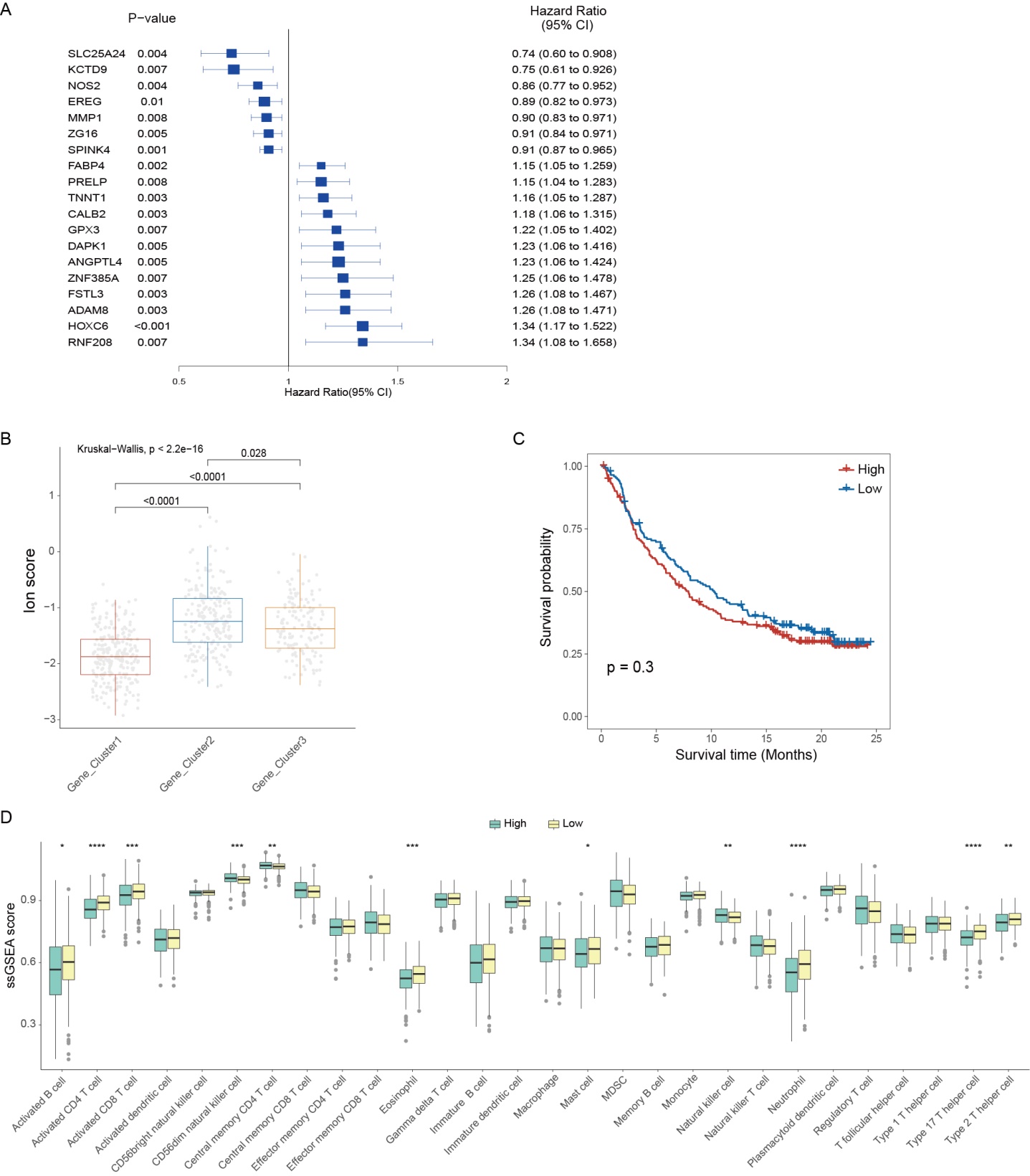

Supplement: Supplementary file 2 — Additional file 2: Figure S1. Flowchart of the study. Figure S2. (A) Forest plot showing 36 ion channel genes significantly associated with prognosis identified by univariate cox regression. (B–D) Indicators of different number of clusters in the consistency clustering method. Figure S3. (A, B) Box plots demonstrating the variations in immunization scores and ESTIMATE scores among ion channel subtypes. (C) The proportion of 22 immune cell subsets in 619 CRC samples. (D) Box plot showing the differences in the proportion of 22 immune cell infiltration among ion channel subtypes. Figure S4. (A) Venn Diagram showing the amount of intersections of differentially expressed genes among each ion channel subtype. (B–D) Indicators of different number of clusters in the consistency clustering based on DEGs. (E) 28 immune cells infiltration abundance of three gene subtypes. (F) Differences in the TIDE scores of gene subtypes. Figure S5. (A) Forest plot demonstrating prognosis-related DEGs among ion channel subtypes. (B) Kaplan–Meier curve showing differences between low- and high-scoring groups in the IMvigor210 cohort. (C) Difference of ion channel score among three Gene clusters in the TCGA. (D) 28 immune cells infiltration abundance in the high- and low-scoring groups. [file 40001_2024_1819_MOESM2_ESM.docx]
